# Gut dysbiosis associates with cytokine production capacity in viral-suppressed people living with HIV

**DOI:** 10.3389/fcimb.2023.1202035

**Published:** 2023-07-31

**Authors:** Yue Zhang, Sergio Andreu-Sánchez, Nadira Vadaq, Daoming Wang, Vasiliki Matzaraki, Wouter A. van der Heijden, Ranko Gacesa, Rinse K. Weersma, Alexandra Zhernakova, Linos Vandekerckhove, Quirijn de Mast, Leo A. B. Joosten, Mihai G. Netea, André J. A. M. van der Ven, Jingyuan Fu

**Affiliations:** ^1^ Department of Genetics, University of Groningen, University Medical Center Groningen, Groningen, Netherlands; ^2^ Department of Pediatrics, University of Groningen, University Medical Center Groningen, Groningen, Netherlands; ^3^ Department of Internal Medicine, Radboud Center for Infectious Diseases, Radboud University Medical Center, Nijmegen, Netherlands; ^4^ Department of Gastroenterology and Hepatology, University Medical Center Groningen, Groningen, Netherlands; ^5^ HIV Cure Research Center, Department of Internal Medicine and Pediatrics, Faculty of Medicine and Health Sciences, Ghent University and Ghent University Hospital, Ghent, Belgium; ^6^ Department of Medical Genetics, Iuliu Hațieganu University of Medicine and Pharmacy, Cluj-Napoca, Romania; ^7^ Department of Immunology and Metabolism, Life and Medical Sciences Institute, University of Bonn, Bonn, Germany

**Keywords:** human gut microbiome, HIV infection, chronic inflammation, cytokine producing capacity, bacterial strain diversity

## Abstract

**Background:**

People living with human immunodeficiency virus (PLHIV) are exposed to chronic immune dysregulation, even when virus replication is suppressed by antiretroviral therapy (ART). Given the emerging role of the gut microbiome in immunity, we hypothesized that the gut microbiome may be related to the cytokine production capacity of PLHIV.

**Methods:**

To test this hypothesis, we collected metagenomic data from 143 ART-treated PLHIV and assessed the *ex vivo* production capacity of eight different cytokines [interleukin-1β (IL-1β), IL-6, IL-1Ra, IL-10, IL-17, IL-22, tumor necrosis factor, and interferon-γ] in response to different stimuli. We also characterized CD4^+^ T-cell counts, HIV reservoir, and other clinical parameters.

**Results:**

Compared with 190 age- and sex-matched controls and a second independent control cohort, PLHIV showed microbial dysbiosis that was correlated with viral reservoir levels (CD4^+^ T-cell–associated HIV-1 DNA), cytokine production capacity, and sexual behavior. Notably, we identified two genetically different *P. copri* strains that were enriched in either PLHIV or healthy controls. The control-related strain showed a stronger negative association with cytokine production capacity than the PLHIV-related strain, particularly for Pam3Cys-incuded IL-6 and IL-10 production. The control-related strain is also positively associated with CD4^+^ T-cell level.

**Conclusions:**

Our findings suggest that modulating the gut microbiome may be a strategy to modulate immune response in PLHIV.

## Highlights

1. We identified compositional and functional changes in the gut microbiome of PLHIV that were strongly related to sexual behavior.
2. PLHIV-associated bacterial changes are negatively associated with HIV reservoir. The relative abundance of both *Firmicutes bacterium CAG 95* and *Prevotella* sp. *CAG 5226* shows a negative association with CD4^+^ T-cell–associated HIV-1 DNA.
3. *Prevotella copri* and *Bacteroides vulgatus* show association with PBMC production capacity of IL-1β and IL-10 that is independent of age, sex, BMI, and sexual behavior.
4. We observed two genetically different *P. copri* strains that are enriched in PLHIV and healthy individuals, respectively.
5. The control-related *P. copri* strain shows a stronger negative association with IL-6 and IL-10 production and a positive association with CD4^+^ T-cell level, suggesting that it plays a potential protective role in chronic inflammation, which may be related to enrichment of a specific epitope peptide.

## 1 Introduction

Human immunodeficiency virus (HIV) infection, as shown in our previous study, induces chronic activation of the innate and adaptive immune systems, leading to a chronic inflammatory state (van der Heijden et al., 2021). Combination antiretroviral therapy (ART) significantly decreases immune activation and systemic inflammation but does not restore the homeostasis in the immune system to that seen in healthy populations (Hileman and Funderburg, 2017). The persistent inflammation, due, in part, to a dysbalanced cytokine system, contributes to a higher risk of non-AIDS–related morbidity in people living with HIV (PLHIV), including cardiovascular disease, neurocognitive disease, and certain HIV-related cancers (Keating et al., 2012; Hileman and Funderburg, 2017). Previous studies have shown that HIV triggers the production of proinflammatory cytokines [tumor necrosis factor (TNF), interleukin-6 (IL-6), and IL-1] and anti-inflammatory cytokines (IL-10) (Osuji et al., 2018; Planès et al., 2018). Starting ART has been shown to decrease plasma concentrations of IL-10 and IL-6, but the concentrations of TNF and other proinflammatory cytokines remain elevated (Osuji et al., 2018). There are multiple potential causes for this dysregulated cytokine system, such as the effect of HIV, lymphoid tissue damage (Klatt et al., 2013), and, in particular, gut dysbiosis (Crakes and Jiang, 2019).

HIV infection induces significant changes in gut microbial composition and metabolic function (Vázquez-Castellanos et al., 2018; Vujkovic-Cvijin and Somsouk, 2019). ART can partially restore the HIV-associated gut dysbiosis, but it cannot normalize the gut microbiome to a pattern resembling that of a healthy control (HC) population (Lozupone et al., 2013; Ray et al., 2021). Compared with HCs, long-term treated PLHIV still exhibit decreased alpha diversity, increased abundances of Enterobacteriaceae, and decreased abundances of *Bacteroidetes* and *Alistipes* (Crakes and Jiang, 2019) and butyrate-producing bacteria that help maintain healthy gut homeostasis (Pinto-Cardoso et al., 2017). However, no consistent pattern of gut dysbiosis has been defined in long-term treated PLHIV (Tuddenham et al., 2020). One major reason for this is that sexual behavior may have a strong influence on gut microbiota, and most previous studies were not able to control for this factor (Tuddenham et al., 2020). For example, an overrepresentation of *Prevotella* accompanied with a decrease in *Bacteroides* in PLHIV was recently found to be due to men who have sex with men (MSM) status rather than HIV infection (Vujkovic-Cvijin et al., 2013; Noguera-Julian et al., 2016; Armstrong et al., 2018; Vujkovic-Cvijin et al., 2020). Considering that a major part of the HIV-infected population in Europe and North America is composed of MSM, the biological importance of this feature needs to be explored.

In long-term treated PLHIV, a link between gut dysbiosis and host cytokine levels has been reported. For instance, in PLHIV with atherosclerosis, class Clostridia was positively correlated with plasma levels of IL-1β and interferon-γ (IFN-γ) (Ishizaka et al., 2021), whereas coproic acid, a gut bacteria–derived short-chain fatty acid (SCFA), was linked with decreased expression of IL-32 (El-Far et al., 2021). Mechanistically, the bacteria–cytokine association is potentially based on bacterial components and products (Dillon et al., 2012; Dillon et al., 2016; Dillon et al., 2017; Vázquez-Castellanos et al., 2018). For example, heat-killed *Escherichia coli* induced a higher production of IL-17 and IFN-γ in HIV-exposed mononuclear cells *ex vivo* (Dillon et al., 2012). In addition, lipopolysaccharide (LPS), a Gram-negative bacterial cell wall component, induced a depletion of CD4^+^ cells by increasing expression of HIV coreceptor C-C chemokine receptor type 5 on CD4^+^ cells (Dillon et al., 2016). In addition, butyrate, a product of saccharolytic fermentation of dietary fibers by gut microbiota, decreased gut T-cell activation in an *ex vivo* human intestinal cell culture model (Dillon et al., 2017). The downregulation of anti-inflammatory bacterial pathways, such as SCFA biosynthesis or indole production, also contributes to gut inflammation in long-term treated PLHIV (Vázquez-Castellanos et al., 2018).

The present study documents a detailed profile of gut microbial composition and function at both species and strain levels using metagenomic sequencing in PLHIV. We then characterize the association of gut dysbiosis in relation to HIV clinical phenotypes and peripheral blood mononuclear cell (PBMC) cytokine production capacity. Notably, the association between cytokine production capacity and gut microbiome has only been studied in healthy populations (Schirmer et al., 2016) and in a limited number of PLHIV (Lozupone et al., 2013). In addition, we control for sexual behavior–related factors in the association analysis. Finally, we identify two *P. copri* strains with different genetic repertoires that exhibit enrichment in PLHIV and HCs, respectively. The control-related *P. copri* strain showed stronger associations with the PBMC production capacity of IL-6 and IL-10, as well as the CD4^+^ T-cell level.

## 2 Methods

### 2.1 Study cohorts

The HIV cohort used in this study was described in our previous study (van der Heijden et al., 2021), in which we recruited 211 PLHIV from the HIV clinic of the Radboud University Medical Center between December 2015 and February 2017. Caucasian individuals who were 18 years of age or older were on combination antiretroviral therapy (cART) for more than 6 months with an HIV-RNA load greater than or equal to 200 copies/mL and showed no signs of opportunistic infections or active hepatitis B/C were included. Venous blood was collected in sterile 10-mL ethylenediaminetetraacetic acid (EDTA) and 8-mL serum BD Vacutainer tubes (Becton Dickinson) and processed within 1–4 h. Isolation of PBMCs and monocytes was performed on freshly collected blood by density centrifugation over Ficoll-Paque (VWR) or by the Pan Monocyte Isolation Kit (Miltenyi Biotec), respectively. Study participants self-collected stool at their homes no more than 24 h prior to study visits, using an empty sterile 50-mL tube with a screwcap with integrated spoon inside. Samples were stored in a refrigerator until being brought in for their visits and then were aliquoted and frozen at −80°C. For this study, 143 metagenomic sequencing samples were available. Participants were excluded if they reported any antibiotics usage in the 3 months prior to fecal sample collection. One fecal sample was analyzed per individual. We included 190 age- and sex-matched HCs from the Dutch Microbiome Project (DMP) cohort as the control group (Gacesa et al., 2022). To replicate our findings in an independent cohort, we also included 173 sex-matched HCs from the 500 Functional Genomics Project (500FG) cohort (Schirmer et al., 2016).

### 2.2 Metagenomic data generation and profiling

The same protocol for fecal DNA isolation and metagenomic sequencing was used for both HIV samples and heathy control samples. Fecal DNA isolation was performed using the QIAamp Fast DNA Stool Mini Kit FSK; Qiagen, cat. no. 51604). The stool samples are lysed using the proteinase K and then heated in the temperature of 95°C. Fecal DNA was sent to Novogene to conduct library preparation and perform whole-genome shotgun sequencing on the Illumina HiSeq platform. We filtered out the low-quality reads by trimming the bases with PHRED quality below 30 and discarded the reads with the length below 70 base pairs after trimming. The reads aligning to human genome were removed by mapping the data to the human reference genome (version HCBI37) using KneadData (v0.7.4). After filtering, the average read depth was 26.8 million for 143 HIV samples, 23.1 million for 190 DMP samples, and 25.1 million for 173 500FG samples. Microbial taxonomic and functional profiles were determined using Metaphlan3 (v3.0.7) (Beghini et al., 2021) and HUMAnN3 (v3.0.0.alpha.3) (Beghini et al., 2021). MetaPhlAn3 is a bioinformatic method to determine microbial composition by mapping reads against a database of nearly 1 million unique clade-specific marker genes identified from around 17,000 reference genomes (13,500 bacterial and archaeal, 3,500 viral, and 110 eukaryotic) (https://huttenhower.sph.harvard.edu/metaphlan3/). The reads identified by MetaPhlAn3 are mapped to species-specific pangenomes with UniRef90 annotations, and the MetaPhlAn3-unclassified reads are translated and aligned to a protein database. Bacteria/pathways present in < 20% of the samples from one cohort were discarded.

### 2.3 Strain profiles and analysis

We used Pangenome-based Phylogenomic Analysis3 (PanPhlAn3) (Beghini et al., 2021) to identify the gene composition at the strain level. A total of 4,998 gene families from the *P. copri* pangenome were detected across 282 samples from the three cohorts. A Jaccard distance matrix was built according to the presence/absence pattern of gene families. The strain cluster tree was constructed using the R basic function *hclust* with the hierarchical clustering method “complete”. Three strain clusters were defined at a tree height of 0.3. Visualizations were generated using the *dendextend* R package. Differentially abundant gene families were obtained using a logistic regression model, controlling for sex, age, and read counts. The subsequent Pfam enrichment analysis was conducted using the *clusterProfiler* R package (v. 3.18.1) (*P. copri* Pfam annotation from PhanPhlan3), where the p-value for enrichment can be calculated by hypergeometric distribution. In the association analysis between cytokine production and HIV-related parameters and the two *P. copri* strains, we used linear regression, controlling for age, sex, read counts, and sexual behavior, as well as using false discovery rate (FDR) < 0.1 as the significant threshold. We identified five *P. copri* peptides with immune function in Immune Epitope Database and Analysis Resource (IEDB) (Vita et al., 2019) (**Supplementary Table 25**) and checked their abundance across the two strains in PLHIV and HCs using ShortBRED (Kaminski et al., 2015) using linear regression and controlling for age, sex, and read counts.

### 2.4 Microbial compositional and differential abundance analysis

The relative abundance data obtained from MetaPhlan3 were used to calculate bacterial diversity using the vegan R package (v. 2.5-7). Alpha diversity was calculated using the *diversity* function. The Bray–Curtis distance n-by-n matrix was built using the *vegdist* function, and, then, the PERMANOVA statistical test was applied on the matrix. We used the principal coordinates analysis method to visualize the dissimilarities of beta diversity between different cohorts. To obtain the differentially abundant bacterial species and pathways between PLHIV and HCs, we first transformed the relative abundance data using centered log-ratio (CLR) transformation, as described before (Gacesa et al., 2022). Bacteria/pathways present in < 20% samples in at least one cohort were then discarded, and the remaining data were inverse-rank–transformed to follow a normal distribution. A linear regression model was then fitted, controlling for body mass index (BMI), smoking status, and read counts. Benjamini–Hochberg correction was used to correct for multiple hypothesis testing (using FDR < 0.05 as the significance threshold). Network of bacterial pathways was conducted using igraph R package (v. 1.2.6), with layout method of “layout_with_fr,” and the bacterial pathways are annotated into super pathways based on Metacyc Database. Dysbiosis index (DI) and function imbalance (FI) scores were calculated as the log2 ratio between geometric means of relative abundances of species/pathways that were enriched in PLHIV (linear regression FDR < 0.05 and HIV cohort beta > 0 in PLHIV vs. HCs) and depleted in PLHIV (linear regression FDR < 0.05 and HIV cohort beta < 0 in PLHIV vs. HCs). DI and FI scores for HCs from the 500FG cohort were calculated using the same species/pathways from the comparison between the HIV and DMP cohorts.

### 2.5 Measurement and analysis of ex vivo PBMC cytokine production, intestinal damage, and monocyte activation

The detailed methods of *ex vivo* PBMC stimulation, cytokines measurements, and plasma markers measurements have been described before (van der Heijden et al., 2021). Venous blood was collected and processed within 1–4 h in sterile 10-mL EDTA and 8-mL serum BD Vacutainer containers (Becton Dickinson). In short, density centrifugation was performed on freshly collected venous blood to obtain the isolation of PBMCs. The freshly isolated cells were then incubated with different bacterial, fungal, and viral stimuli at 37°C and 5% CO_2_ for either 24 h or 7 days. IL-1β, IL-6, IL-1Ra, IL-10, and TNF were determined in the supernatants of the 24-h PBMC or monocyte stimulation experiments using enzyme-linked immunosorbent assay (ELISAs). IL-17, IL-22, and IFN-γ were measured after the 7-day stimulation of PBMCs. Cytokine production data for the 500FG cohort were obtained using the same method, and the measurements that overlapped with those in the HIV cohort are summarized in **Supplementary Table 9**. For the comparison of cytokine production between PLHIV and HCs from 500FG, we used different samples compared with the previous studies (van der Heijden et al., 2021; Van de Wijer et al., 2021), so we conducted a reanalysis. A linear regression model was used, with correction for sex and age, using FDR < 0.05 as the significant threshold. Differentially abundant cytokine production and eight kinds of anti-inflammatory cytokine production (IL-10 and IL-1Ra) were included in the subsequent analysis. Furthermore, in the HIV cohort, the plasma levels of intestinal fatty acid–binding protein (iFABP), a marker of microbial translocation, and monocyte activation markers (sCD14 and sCD163) were measured using ELISA (Duoset or Quantikine, R&D Systems).

### 2.6 Microbial associations to HIV-related variables, cytokine production capacity, intestinal damage, and monocyte activation

For association analysis between gut microbiome and HIV-related variables, cytokine production capacity, intestinal damage, and monocyte activation, we included bacterial alpha diversity (Shannon index), beta diversity, *Prevotella*-to-*Bacteroides* (P/B) ratio, DI and FI scores, as well as 76 species and 163 pathways that were significantly different between PLHIV and HCs from DMP cohort. Before Spearman correlation analysis, we first inverse-rank–transformed the data to follow a standard normal distribution and then adjusted the bacteria–phenotype associations for confounding factors (age and sex) and a technical variable (read counts). Significant associations were defined at FDR < 0.1 level. In additional analyses, we further adjusted for sex behavior factors [sexual orientation (SO) and the number of sexual partners in the previous year (Num-P)].

## 3 Results

### 3.1 Microbial dysbiosis in PLHIV

#### 3.1.1 Differences in microbial composition and function

**Figure 1:**
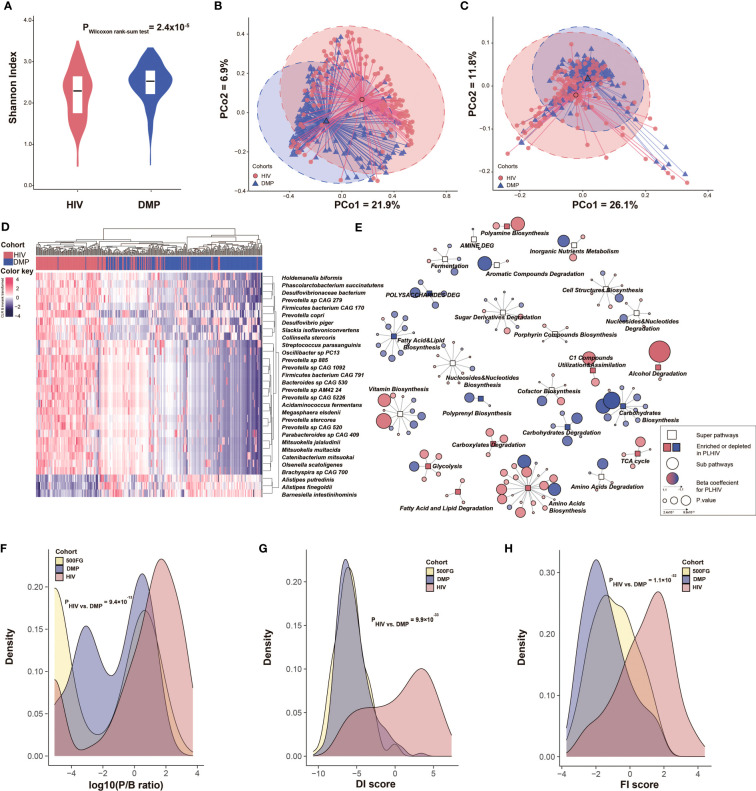
PLHIV show a distinct gut microbiome composition and function compared with HCs. **(A)** Comparison of microbial alpha diversity between the PLHIV cohort and HCs from the DMP cohort. Y-axis refers to the Shannon index at the species level. **(B, C)** Beta diversity based on Bray–Curtis distance of species and pathway abundance is shown in a principal coordinates analysis (PcoA) plot with centroids for PLHIV and HCs. The coordinates of the centroids are set as the mean value of the principal components for each cohort. **(D)** Heatmap depicting the relative abundance of the top 30 species that differed significantly between PLHIV and HCs. Data are CLR-transformed and then inverse-rank–transformed to follow a normal distribution. Differentially abundant species were selected using a linear regression model with correction for BMI, read counts, and smoking status. **(E)** Network of bacterial pathways that were significantly different between PLHIV and HCs. Rectangular nodes represent super-pathways, with the colors showing enrichment (pink) or depletion (blue) in PLHIV. Circular nodes show sub-pathways belonging to the super-pathways. The color of circles shows the log2 value of fold change between the relative abundance of pathway in PLHIV and HCs, where a gradient is applied depending on fold change. Circle size indicates p-value. Lines connect each pathway to its respective super-pathway. Only super-pathways including two or more pathways and sub-pathways with FDR < 0.05 are shown. Differentially abundant pathways were selected using a linear regression model with correction for BMI, read counts, and smoking status. **(F–H)** Density curves of the P/B ratio, DI score, and FI score for the three cohorts depicting the different distribution of these bacterial signatures in these cohorts. Significance was tested using Dunn’s test.

The present study included 143 PLHIV and 190 healthy individuals with matched age and sex from the DMP cohort (hereafter referred to as matched HCs). Participants’ baseline characteristics are shown in **Table 1**. Included PLHIV were on long-term ART (median of 6.35 years) and were virally suppressed (plasma HIV-RNA < 200 copies/mL). However, when compared with the matched HCs, the gut microbial composition of the PLHIV still showed a significant decrease in alpha diversity (species-level Shannon index, Wilcoxon rank sum test, p = 2.4 × 10^−5^; **Figure 1A**) and a distinct composition that was reflected by a significant difference in beta diversity (PERMANOVA, p < 1.0 × 10^−3^, R^2^ = 0.07; **Figure 1B**), even when adjusting for BMI, read counts, and smoking status. These observations are consistent with findings of previous studies (Nowak et al., 2015; Zilberman-Schapira et al., 2016; Gootenberg et al., 2017). With the aid of metagenomics data, we also observed that PLHIV showed a lower diversity of bacterial metabolic pathways (pathway-level Shannon index, Wilcoxon rank sum test, p = 4.0 × 10^−4^; **Supplementary Figure 1**) and different functional profiles (PERMANOVA, p < 1.0 × 10^−3^, R^2^ = 0.04; **Figure 1C**). To explore the bacterial taxa and pathways that showed significantly different abundance in PLHIV, we confined the differential abundance analysis to the 123 common species and 331 metabolic pathways present in ≥ 20% of samples in at least one cohort. A linear regression model with correction for read counts, BMI, and smoking status revealed 76 (62.6%) species and 163 pathways (49.2%) with differential abundances between PLHIV and HCs (FDR < 0.05; **Supplementary Tables 1, 2**). Notably, sexual behavior is a known factor that influences the gut microbiome (Tuddenham et al., 2020), and the PLHIV was enriched for c (MSM) (n = 96, 67%). We further included a control group of 53 men from the DMP cohort who reported to have male partners (DMP-MSM) during sample collection time (**Table 1**). Of the 76 differentially abundant species and 163 pathways, 51 species (89.5%) and 102 pathways (62.6%) were replicated at FDR < 0.05 level (**Supplementary Tables 3, 4**, **Supplementary Figures 2, 3**), suggesting that most of our reported differences between PLHIV and healthy individuals were not cofounded by sex behavior.

**Table 1:** Baseline characteristics of study populations.

Baseline characteristics	PLHIV vs. healthy controls with matched age and gender			PLHIV vs. healthy controls with matched sexual behavior	
	PLHIV from HIV cohort	Selected HCs from DMP cohort with matched age and gender	Selected HCs from 500FG cohort with matched gender	MSM from HIV cohort	MSM from DMP cohort
Sample size	143	190	173	96	53
Sex (male/female)	130/13	166/24	158/15	96/0	53/0
No. of MSM (%)	96 (67%)	1 (0.5%)	–	96 (100%)	53 (100%)
No. of MSW (%)	24 (12.6%)	165 (86.8%)	–	–	–
No. of individuals with RAI last year (%)	35 (24.5%)	–	–	35/96 (36.5%)	0
Age [years, mean (SD)]	51.5 (10.7)	50.7 (11.1)	30.4 (15.7)	50.7 (10.7)	49.5 (12.4)
BMI [kg/m^2^, mean (SD)]	24.5 (3.50)	25.5 (3.14)	23.2 (2.63)	24.0 (3.2)	25.5 (3.4)
No. of current smoking (%)	40 (28.0%)	19 (10.0%)	23 (12.9%)	24 (25%)	3 (5.7%)
Duration of HIV infection [years, median (IQR)]	8.1 (5, 13.5)	–	–	7.6 (4.8, 12.7)	–
Duration of cART [years, median (IQR)]	6.4 (4.1, 12)	–	–	6.3 (4.1,10.0)	–
CD4 nadir [10^6^ cells/mL, median (IQR)]	260 (120, 370)	–	–	275 (190, 380)	–
CD4 count (10^6^ cells/mL, median (IQR)]	650 (465, 810)	–	–	635 (500, 802.5)	–
HIV RNA load in plasma below 200 copies/mL [n (%)]	143 (100%)	–	–	96 (100%)	–
Diabetes (%)	10 (7.0%)	–	–	4 (4.2%)	–
Hypertension (%)	27 (18.9%)	–	–	19 (19.8%)	–
Hypercholesterolemia (%)	38 (26.6%)	–	–	19 (19.8%)	–
Metal problems (%)	35 (24.5%)	–	–	25 (26.0%)	–
Malignancies (%)	20 (14.0%)	–	–	12 (12.5%)	–

Heavy drinking (for men, ≥15 drinks/week; and for women, ≥8 drinks/week).

BMI, body mass index; RAI, receptive anal intercourse; DMP cohort: Dutch Microbiome Project; 500FG cohort: 500 Functional Genomics; HCs, healthy controls; Project; PLHIV, people living with human immunodeficiency virus; MSM, men who have sex with men; MSW, men who have sex with women; SD, standard deviation; cART, combination antiretroviral therapy; IQR, interquartile range.

-, data is not available.

#### 3.1.2 Differentially abundant species

Fifty-seven of the 76 differentially abundant species showed enrichment in PLHIV. Increased abundances of *Prevotella* and *Prevotellaceae* in PLHIV were widely observed by previous studies using 16S ribosomal RNA (rRNA) sequencing (Vujkovic-Cvijin and Somsouk, 2019). Our metagenomics-based analysis further identified eight *Prevotella* species enriched in PLHIV (**Supplementary Table 1**). The most significant was *Prevotella* sp. *885*, which showed a 3.8-fold increase in relative abundance (linear regression, p = 2.1 × 10^−27^), followed by *Prevotella* sp. *CAG 520* with an 8.0-fold increase (p = 5.4 × 10^−24^) and *Prevotella* sp. *CAG 1092* with a 4.2-fold increase (p = 3.4 × 10^−23^; **Figure 1D**, **Supplementary Figure 4**). Also consistent with other studies (Lozupone et al., 2013; Vázquez-Castellanos et al., 2015), we found an increase of *Desulfovibrionaceae bacterium* (p = 7.8 × 10^−24^) and *Megasphaera elsdenii* (p = 3.9 × 10^−23^). *Megasphaera* species, as members of the vaginal microbiome, were associated with a higher risk of acquiring HIV in a prospective study of HIV-infected South African women (Gosmann et al., 2017). The abundances of 19 species were decreased in PLHIV, including species from the previously reported species *Bacteroides* and *Alistipes* (Crakes and Jiang, 2019; Vujkovic-Cvijin and Somsouk, 2019): *B. ovatus*, *B. uniformis*, *B. vulgatus*, *A. finegoldii*, and *A. putredinis*. We also identified several novel HIV-associated species, including *Barnesiella intestinihominis*, which was mostly depleted in PLHIV (p = 7.5 × 10^−15^). This bacterium was previously identified as an “oncomicrobiotic” due to its capacity to promote the infiltration of IFN-γ–producing γδT cells in cancer lesions, which can ameliorate the efficacy of the anti-cancer immunomodulatory agent cyclophosphamide (Daillère et al., 2016).

#### 3.1.3 Differentially abundant pathways

At the metabolic pathway level, we observed differential abundances in several amino acid biosynthesis pathways, including enriched L-tryptophan biosynthesis (PWY-6629) and depleted L-ornithine and L-citrulline biosynthesis pathways (ARGININE-SYN4-PWY and CITRULBIO-PWY) in PLHIV (**Supplementary Table 2**). Importantly, both tryptophan and citrulline play critical roles in inflammation (Murray, 2003; Papadia et al., 2010), whereas ornithine can later be turned into nitric oxide (NO), which is important for vascular function (Baumgartner et al., 2000; Dirajlal-Fargo et al., 2017). In addition, the reductive tricarboxylic acid (TCA) cycle I (P23-PWY) was enriched in PLHIV. The reductive TCA cycle is a carbon dioxide–fixation pathway significant for the production of organic molecules for the biosynthesis of sugars, lipids, amino acids, and pyrimidines (Smith and Morowitz, 2004). Moreover, after clustering bacterial pathways to the same biochemical processes, we found that PLHIV showed lower abundances of bacterial pathways involved in fatty acid, lipid, and carbohydrate biosynthesis but higher abundances of bacterial pathways involved in amino acids biosynthesis and TCA cycle (**Figure 1E**).

#### 3.1.4 Dysbiosis index

Together, our data show dysbiosis in both gut microbial composition and metabolic function in PLHIV. Previous studies have suggested the P/B ratio as a landmark parameter for PLHIV (Dillon et al., 2014; Ling et al., 2016), and our finding of a significantly higher P/B ratio in PLHIV compared with HCs confirms these observations (Dunn’s test, p = 9.4 × 10^−13^; **Figure 1F**, **Supplementary Table 5**, **Supplementary Figure 5A**). We also constructed a DI based on the differentially abundant species by calculating the log2 ratio of the geometric mean of PLHIV-enriched species (57 species) to PLHIV-depleted species (19 species). This DI score was significantly higher in PLHIV than in HCs (p = 9.9 × 10^−33^; **Figure 1G**, **Supplementary Table 5**). We then sought to validate the DI score in an independent cohort with similar metagenomic data and the same DNA isolation method. Whereas no such data were available for an independent cohort of PLHIV, data whereas available for a separate healthy cohort: 500FG (**Table 1**). We found that the DI score of 500FG was not different from the DMP controls (p = 0.29; **Supplementary Figure 5B**) but was significantly lower than that of our cohort of PLHIV (p = 9.6 × 10^−37^). We also constructed a FI score using the log2 ratio of the geometric mean of PLHIV-enriched bacterial pathways (87 pathways) to PLHIV-depleted bacterial pathways (76 pathways) (**Figure 1H**). This FI score was also significantly higher in PLHIV than in DMP HCs (p = 1.1 × 10^−32^; **Supplementary Table 5**, **Supplementary Figure 5C**), and this was supported by the observation that the 500FG FI was also significantly lower than that of PLHIV (p = 6.7 × 10^−18^).

### 3.2 HIV-associated gut dysbiosis associates with clinical phenotypes

We conducted a systematic association analysis between HIV-related variables and microbial alpha diversity, beta diversity, P/B ratio, DI score, and FI score, correcting for sex, age, and read counts (**Supplementary Figures 6, 7**, **Supplementary Table 6**). HIV clinical parameters were also taken into account, including the time between HIV diagnosis and inclusion in the study or cART initiation, CD4^+^ T-cell counts (nadir and latest, recovery after cART), plasma viral loads, and HIV-1 reservoir measurements in circulating CD4^+^ T cells, including the CD4^+^ T-cell–associated HIV-1 DNA (CA-HIV-DNA) and CD4^+^ T-cell–associated HIV-1 RNA (CA-HIV-RNA) levels. Sexual behavior was also considered part of the HIV clinical phenotype, including Num-P and SO, e.g., MSM and receptive anal intercourse (RAI).

**Figure 2:**
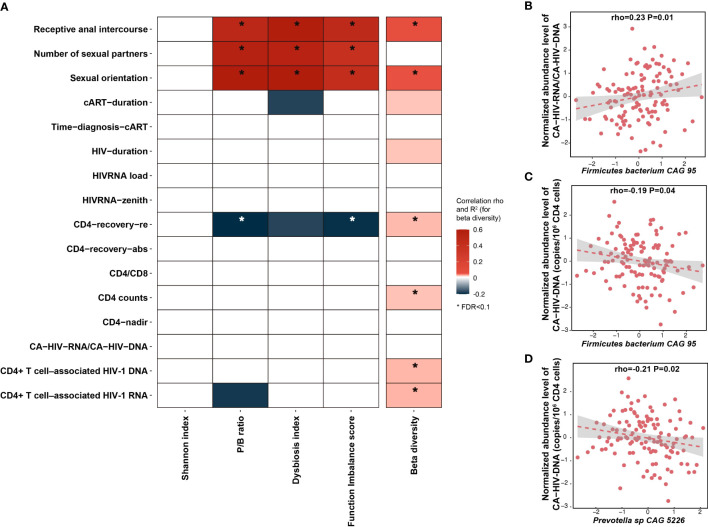
Association between HIV-associated gut dysbiosis and HIV-related variables. **(A)** Heatmap depicting the associations between HIV-associated bacterial signature (Shannon index, beta diversity, P/B ratio, DI score, and FI score) and HIV-related variables, using the Spearman correlation test, with correction for age, sex, and read counts. Box color indicates Spearman correlation rho if the association p-value < 0.05. The associations at FDR < 0.1 level are highlighted with *. R^2^ is calculated using PERMANOVA based on Bray–Curtis distance of species and then multiplied by 10 to rescale. **(B–D)** Associations between individual species and HIV reservoir level, with correction for age, sex, read counts, and sexual behavior.

We did not observe any significant association between the Shannon index of bacterial species and any HIV clinical phenotypes (**Supplementary Table 7**). However, six parameters were associated with bacterial beta diversity, four with P/B ratio, three with DI score, and four with FI score at FDR < 0.1 level (**Figure 2A**, **Supplementary Table 7**). Notably, although most of the reported microbial differences between PLHIV and HCs were not driven by sexual behavior (**Supplementary Tables 3, 4**), within PLHIV, sex behavior was still among the strongest associated factors. For example, SO was the top factor positively associated with the P/B ratio (Spearman correlation, p = 5.7 × 10^−12^) and DI score (p = 6.4 × 10^−12^), followed by Num-P and RAI (**Supplementary Table 7**). Moreover, MSM and RAI, as well as larger Num-P, were associated with increased P/B ratio, DI score, and FI score. We also observed associations for other HIV clinical phenotypes, such as an association between beta diversity and the HIV reservoir parameters CA-HIV-DNA and CA-HIV-RNA levels (p = 6.0 × 10^−3^ and 8.0 × 10^−3^, respectively) and an association between P/B ratio and CD4 recovery relative rate (p = 0.02). However, these associations were largely dependent on sexual behavior, as none remained significant after correcting for SO and Num-P (**Supplementary Table 7**, **Supplementary Figure 8**). We further assessed whether HIV clinical parameters were associated with individual species and metabolic pathways. After correcting for age, sex, read counts, and sexual behavior, no significant associations were detected for species or pathways, although we did observe suggestive associations for *Firmicutes bacterium CAG 95* and *Prevotella* sp. *CAG 5226* with HIV reservoir measurements at a normal significance level (**Figures 2B–D**, **Supplementary Table 8**). At the metabolic pathway level, HIV duration and cART duration tended to be the strongest factors in addition to sexual behavior factors linked with metabolic pathways (**Supplementary Table 9**). For example, the pathways of stearate, octanoyl, and oleate biosynthesis showed a positive correlation with HIV duration, whereas the top result for cART duration was a negative association with the polyamine biosynthesis pathway (**Supplementary Table 9**). We did not observe any significant associations between the gut microbiome and cART regimens after correcting for sexual behavior (**Supplementary Tables 10, 11**).

### 3.3 Prevotella copri and Bacteroides vulgatus associate with cytokine production capacity

**Figure 3:**
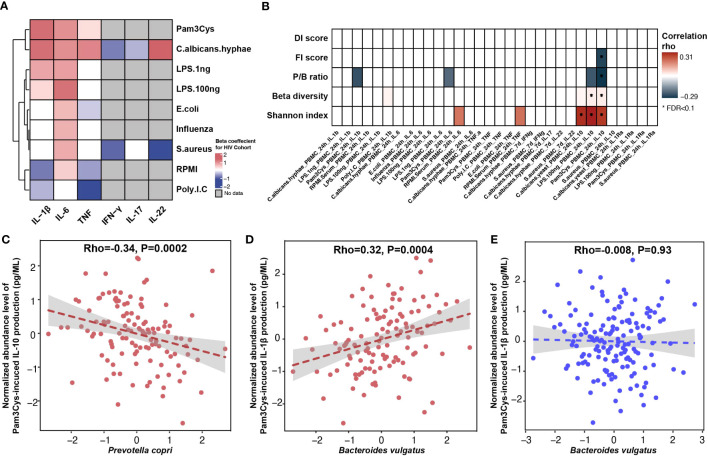
Association between gut microbiome and inflammatory cytokine production. **(A)** Heatmap showing *ex vivo* cytokine production enriched (red) and depleted (blue) in PLHIV as compared with HCs from 500FG. A linear regression model (age and sex included as covariates) was used to calculate the P-values. Box color indicates the Spearman correlation rho for those associations that were significant at p-value < 0.05 level. The associations at FDR < 0.1 level were highlighted with *. White box indicates associations with p-value > 0.05, and gray box indicates no measurement. **(B)** Heatmap showing Spearman correlation rho between cytokine production and HIV-associated bacterial signature (Shannon index, beta diversity, P/B ratio, and DI and FI scores), with correction for age, sex, read counts, and sexual behavior. White box indicates P > 0.05. The row labels indicating the cytokine produced and the name of stimulation. **(C, D)** Association between relative abundance of species and cytokine production in PLHIV: **(C)** *Prevotella copri* with Pam3Cys-induced IL-10 production and **(D)** *Bacteroides vulgatus* with Pam3Cys-induced IL-1β production. **(E)** Association between relative abundance of *Bacteroides vulgatus* and Pam3Cys-induced IL-1β production in HCs from 500FG. The relative abundance of the species was CLR- and inverse-rank–transformed. Association was corrected for age, sex, read counts, and sexual behavior using linear regression. Pam3Cys, synthetic TLR2 ligand; LPS.1ng, PBMC cytokine production in response to LPS (1 ng/mL) stimulation; LPS.100ng, PBMC cytokine production in response to LPS (100 ng/mL) stimulation; RPMI, serum-free medium; Poly IC, TLR3 ligand.

Despite the long-term cART, the immune responses of PLHIV are known to be different than those of HCs (Van de Wijer et al., 2021). For instance, our previous study reported a significant increase in the production of proinflammatory cytokines in PLHIV (van der Heijden et al., 2021). In the present study, we newly compared the cytokine production data of 143 PLHIV (van der Heijden et al., 2021), for whom we collected microbiome data for this study, to 173 HCs from the 500FG cohort for whom the cytokine production capacity was measured previously (Schirmer et al., 2016) (**Supplementary Table 12**). We identified 24 cytokine abundances that were significantly different between PLHIV and healthy population (**Figure 3A**, **Supplementary Table 13**). PLHIV showed a significant increase of IL-1β, IL-6, and TNF production upon stimulation with Pam3Cys (TLR2 ligand), LPS (TLR4 ligand), and *C. albicans hyphae* but decreased IFN-γ production upon stimulation with *S. aureus* and *C. albicans hyphae*. Moreover, cytokine production capacities were also related to some HIV-related phenotypes. MSM status was associated with increased proinflammatory cytokine responses (e.g., Pam3Cys-induced TNF and *S. aureus*–induced IL-22 production), whereas higher Num-P was linked with decreased anti-inflammatory cytokine responses (e.g., Pam3Cys- and LPS-induced IL-10 production; **Supplementary Figure 9**, **Supplementary Table 14**).

Interestingly, we observed significant associations of cytokine production capacity with microbial alpha and beta diversity and P/B ratio, as well as four associations with DI and FI scores and individual species (**Supplementary Tables 15, 16**). No associations were observed with metabolic pathway abundance (**Supplementary Table 17**). In particular, IL-10 production upon stimulation with Pam3Cys or LPS was negatively associated with P/B ratio and FI score but positively associated with bacterial Shannon index (linear regression, p < 0.05; **Figure 3B**, **Supplementary Table 15**), after correction for age, sex, and sexual behavior. At the individual species level, the top association was between Pam3Cys-induced IL-10 production and the relative abundance of *P. copri* (rho = −0.37, p = 9.1 × 10^−6^; **Supplementary Table 16**), after correcting for age, sex, and read counts. After further adjustment for SO and Num-P, the association between *P. copri* and IL-10 production remained significant (rho = −0.34, p = 1.9 × 10^−4^; **Figure 3C**, **Supplementary Table 16**). In addition, we also detected a positive significant association between *B. vulgatus* and Pam3Cys-induced IL-1β production (rho = 0.33, p = 7.4 × 10^−5^), and this remained significant after controlling for age, sex, read counts, and sexual behavior (rho = 0.32, p = 3.7 × 10^−4^; **Figure 3D**). Notably, this association was not significant in 500FG (rho = −7.8 × 10^−3^, p = 0.93; **Figure 3E**), showing a significant heterogeneity effect (Cochran’s Q-test, p = 0.003; **Supplementary Table 16**).

### 3.4 Prevotella copri strains in PLHIV are genetically different

**Figure 4:**
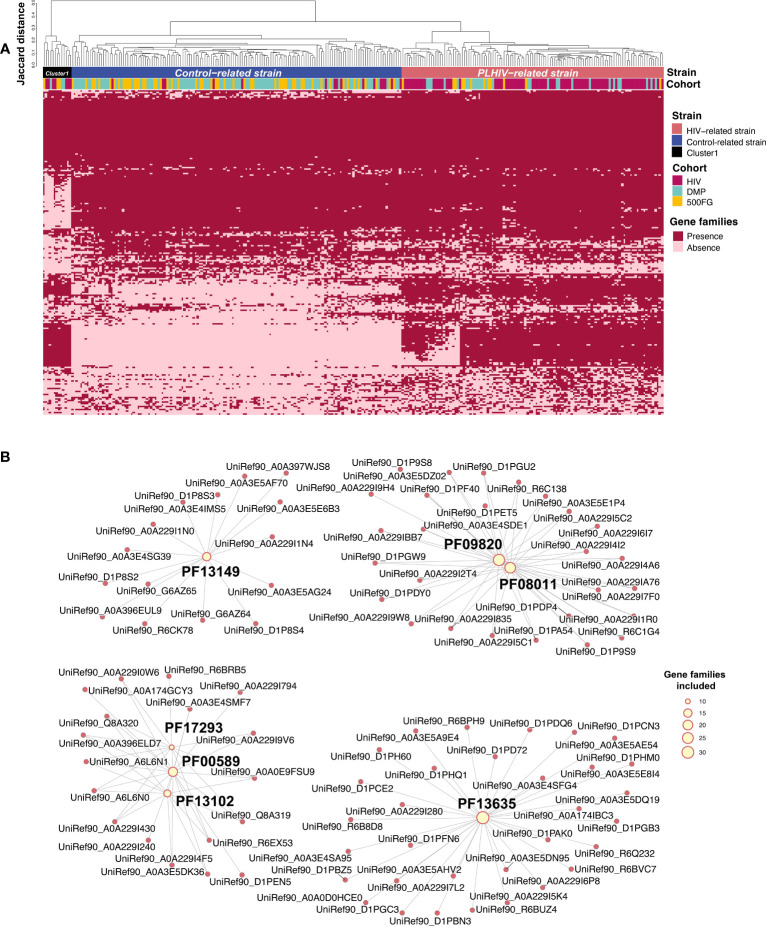
*Prevotella copri* strains with different genetic content. **(A)** Heatmap showing the gene family profiles of *P. copri* strains in samples from the three cohorts (282 samples total: 102 PLHIV, 111 DMP, and 69 500FG). Each column represents a sample. Each row represents the presence or absence of a gene family. The clustered tree above the heatmap shows the three clusters of *P. copri* strains. Most samples from PLHIV were binned together into the PLHIV-related strain (right), but 16 PLHIV samples (middle) showed different profiles and were binned into the control-related strain. **(B)** Network figure showing that the gene families increased in PLHIV-related strain are enriched in seven Pfams (FDR < 0.05). Pink dots represent different gene families. Yellow dots represent Pfams, and their sizes indicate how many gene families annotated to themselves. Lines indicate that the gene families are annotated to the corresponding Pfam. PF00589, phage integrase family; PF13102, phage integrase SAM-like domain; PF17293, Arm DNA-binding domain.

In microbial species, strain-level genomic makeup is critical in determining their functional properties within human bodies (Scholz et al., 2016). We therefore wondered whether PLHIV harbor different strains of *P. copri* and *B. vulgatus*, thereby affecting cytokine production capacity. To examine this, we performed an analysis on the basis of the presence or absence of their gene repertoire using PanPhlan3 (Beghini et al., 2021). As *B. vulgatus* did not show any distinct clusters according to genetic content, we did not study it further. We generated *P. copri* genetic repertoire profiles for 282 samples from the PLHIV cohort (n = 102), DMP cohort (n = 111), and 500FG cohort (n = 69; **Supplementary Table 18**). Hierarchical clustering analysis based on the Jaccard distance of the presence or absence of gene families revealed three distinct clusters at a distance cutoff of 0.3 (**Figure 4A**). Cluster 1 was relatively small (n = 13) and was therefore excluded in the following analysis. In contrast, clusters 2 and 3 were larger (n = 150 and 119, respectively; **Supplementary Table 19**). Interestingly, cluster 2 was significantly enriched in healthy individuals, and cluster 3 was enriched in PLHIV (Fisher exact test, p = 3.7 × 10^−22^; **Supplementary Table 19**). We hereafter refer to these two distinct *P. copri* strains as the “control-related strain” and “PLHIV-related strain,” respectively. We further assessed the difference in the gene content of the two strains. A total of 2,821 of the 4,998 gene families were differentially abundant (logistic regression, FDR < 0.05; **Figure 4A**, **Supplementary Table 20**), and seven protein families (Pfam) (Finn et al., 2010) were enriched in the PLHIV-related strain (hypergeometric test, FDR < 0.05; **Figure 4B**, **Supplementary Table 21**): Three Pfams are related with DNA cleaving and binding, including phage integrase family (PF00589), phage integrase SAM-like domain (PF13102), and Arm DNA-binding domain (PF17293). In contrast, no Pfams showed enrichment in the control-related strain (**Supplementary Table 22**).

Interestingly, the frequency of different *P. copri* strains was also associated with sex behavior, and PLHIV-related strain showed an enrichment in PLHIV-MSM than that in PLHIV-MSW (Fisher exact test, p = 0.01). However, after controlling for the behavior of MSM, this strain was still enriched in PLHIV (Fisher exact test, PLHIV-MSM vs. DMP-MSM, p = 1.1 × 10^−4^; **Supplementary Table 19**, **Supplementary Figure 10**).

### 3.5 Prevotella copri strain profile is associated with cytokine production capacity

**Figure 5:**
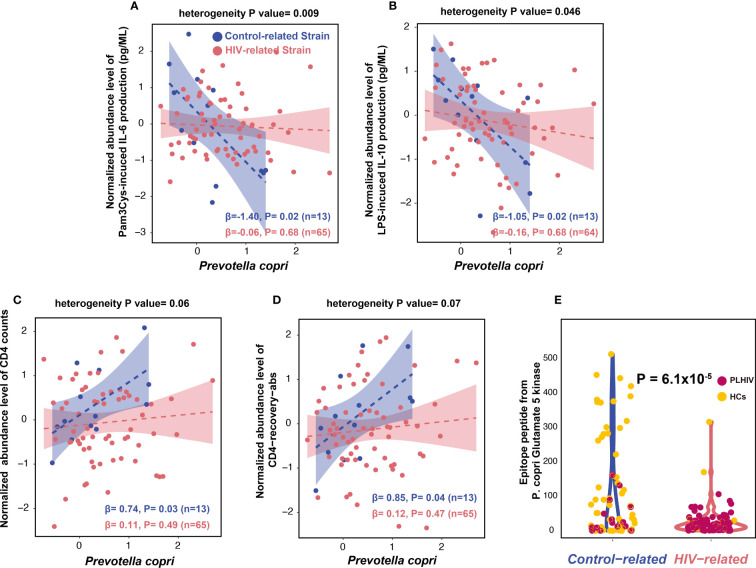
*Prevotella copri* strains with different immune functions. **(A, B)** Distinct associations between control-related strain and PLHIV-related strain with IL-6 and IL-10 production capacity in PLHIV, using linear regression. Association is corrected for age, sex, read counts, and sexual behavior. Blue dots represent the PLHIV with the control-related strain. Dark pink indicates PLHIV with the PLHIV-related strain. Bacterial relative abundance is CLR- and inverse-rank–transformed. **(C, D)** Distinct associations between relative abundance of control-related strain and PLHIV-related strain with CD4 counts **(C)** and CD4-recovery-abs level **(D)**. **(E)** Violin plot of the *P. copri* epitope peptide (from glutamate 5 kinase) level between the two kinds of strains in PLHIV and HCs from the 500FG cohort.

We further compared the associations of different *P. copri* strains with cytokine production capacity. The control-related strain showed stronger associations with cytokine production than PLHIV-related strain, especially for Pam3Cys-induced IL-6 and IL-10 production in PLHIV (**Figures 5A, B**, **Supplementary Table 23**). However, in the 500FG cohort, the control-related strain showed an opposite association with IL-6 production (**Supplementary Figure 11A**, **Supplementary Table 24**). In contrast, PLHIV-related strain also showed a negative association with IL-10 production in response to *Candida albicans* in PLHIV; however, the association did not show heterogeneity against control-related strain (**Supplementary Figure 11B**, **Supplementary Table 23**).

We also checked the association between *P. copri* strains and HIV-related parameters, as well as with monocyte activation markers (sCD14 and sCD163) and microbial translocation marker (IFABP). The control-related strain showed a positive association with CD4^+^ T-cell counts (CD4 counts) and CD4^+^ T-cell absolute recovery level after cART (CD4-recovery-abs) (**Figures 5C, D**, **Supplementary Table 25**). These strain–CD4 associations did not show heterogeneity between the control- and PLHIV-related strains (**Supplementary Table 25**). However, we did not observe any significant associations between *P. copri* strains and monocyte activation and microbial translocation markers (**Supplementary Table 26**).

To explore the potential mechanism behind the distinct immune functions of the two *P. copri* strains, we searched the IEDB and found five epitope peptides derived from different *P. copri* proteins (**Supplementary Table 27**). These peptides can be presented by antigen-presenting cells and induce IFN-γ production by T cells and antibody secretion by B cells. We then compared the abundances of these five peptides between the two *P. copri* strains from PLHIV and 500FG cohort (Methods). Interestingly, only one peptide from *P. copri*, glutamate 5-kinase protein, was found to be significantly enriched in the samples with the control-related strain as compared with the samples with the PLHIV-related strain (linear regression, p = 6.1 × 10^−5^; **Figure 5E**, **Supplementary Table 28**), suggesting that it is potentially contributing to the immune function of the control-related strain.

## 4 Discussion

We performed metagenomics-based microbiome associations of the gut microbiome with *ex vivo* cytokine production capacity in PLHIV. First, we observed a remarkable microbial dysbiosis in PLHIV. The microbial diversity was significantly lower, and the abundance levels of 76 species and 163 metabolic pathways were significantly different from HCs. Sex behavior was identified as an important factor that could influence the gut microbiome, but sex behavior could not explain all the PLHIV-associated microbial differences. Second, PLHIV-associated microbial dysbiosis was associated with clinical parameters and cytokine production capacity in PLHIV, including HIV reservoir, CD4 recovery relative rate, and production capacity of IL-10 and IL-1β. Interestingly, *P. copri* species in PLHIV and HCs showed distinct genetic differences, suggesting that two different strains were present in PLHIV and HCs, respectively. We also found that the control-related *P. copri* strain showed a stronger negative association with IL-6 and IL-10 production and a positive association with CD4 counts.

In the comparison of gut microbial composition between PLHIV and HCs, we observed a lower alpha diversity and higher P/B ratio in PLHIV (**Figures 1A, F**), as well as a depletion of *Alistipes* species (**Figure 1D**) (Crakes and Jiang, 2019; Vujkovic-Cvijin and Somsouk, 2019). In contrast to previous studies, we found some SCFA-producing species and beneficial species to be enriched in PLHIV in our study, including *Acidaminococcus fermentans* (Chang et al., 2010) and *Faecalibacterium prausnitzii* (Parada Venegas et al., 2019), as well as the equol-forming species *Slackia isoflavoniconvertens* (Schröder et al., 2013) and the anti-tumorigenic species *Holdemanella biformis* (Zagato et al., 2020). This inconsistency may be due to the fact that the PLHIV in our cohort had a longer duration of ART intake (median of 6.4 years) compared with previous studies, which is supported by earlier evidence that ART can partially restore the gut microbial composition (Lozupone et al., 2013). Functionally, PLHIV in our study showed an increased microbial capacity of L-tryptophan biosynthesis and a decreased *de novo* biosynthesis of ornithine from 2-oxoglutarate, functions that have been related to inflammation and vascular function (Murray, 2003; Dirajlal-Fargo et al., 2017), respectively. This observation supports the idea that those functional changes in the gut microbiome in PLHIV contribute to the persistent inflammation seen in these individuals. Importantly, we also showed that sex orientation can influence the gut microbiome but the microbial dysbiosis between PLHIV and HCs is not driven by sex orientation only.

Infected long-lived memory CD4^+^ T cells make up the majority of cell types constituting the HIV reservoir (Churchill et al., 2016). To the best of our knowledge, no other studies have ever reported associations between the microbiome and the HIV reservoir. We analyzed HIV-1 DNA and RNA levels in isolated circulating CD4^+^ T cells, which reflect the size of the HIV reservoir (Rutsaert et al., 2019). We observed that the CD4^+^ T-cell counts were significantly associated with bacterial beta diversity (**Figure 2A**). We also found that *Firmicutes bacterium CAG 95* negatively correlated with CA-HIV-DNA levels and that the relation between *R. lactatiformans* and CA-HIV-RNA level was positive, whereas *Prevotella* species showed negative associations with CA-HIV-DNA and CA-HIV-RNA (**Figures 2B–D**). On one hand, how these bacterial species modulate the HIV reservoir remains speculative. *R. lactatiformans* has been linked with increased colonic IFN-γ^+^ T cells and immune activation (Frankel et al., 2019), whereas a decreased abundance of *Firmicutes bacterium CAG 95* was found in subjects with hepatic steatosis (Zeybel et al., 2022). A previous study identified several immunogenic human leukocyte antigen (HLA) – DR isotype – presented *P. copri* peptides with the ability to induce T cells to produce the anti-viral cytokine IFN-γ (Kak et al., 2018; Pianta et al., 2021). In our study, the *P. copri* HC-related strain was associated with decreased levels of IL-6 production, which can facilitate HIV-1 replication (Connolly et al., 2005). On the other hand, HIV infection leads to a severe gastrointestinal (GI) CD4^+^ T cells loss, which may affect immunological tolerance to specific gut microbes and thereby result in bacterial changes (Crakes and Jiang, 2019). This is supported by previous evidence that PLHIV with CD4 counts < 350 cells/mm^3^ for 2 years of cART showed an enrichment of *unclassified Subdoligranulum species* and *Coprococcus comes*, compared with those with CD4 counts ≥ 350 cells/mm^3^ (Lu et al., 2018). Importantly, the relative abundances of unclassified *Subdoligranulum species* and *C. comes* were positively correlated with CD8^+^HLA-DR^+^ T-cell count and CD8^+^HLA-DR^+^/CD8^+^ percentage in PLHIV (Lu et al., 2018). However, we did not observe differences in bacterial composition between PLHIV with CD4 counts ≥ 500 cells/mm^3^ and PLHIV with CD4 counts < 500 cells/mm^3^. Moreover, the HIV reservoir was also correlated with CD4 counts, HIV infection, and treatment duration (**Supplementary Figure 7**). Therefore, the causal direction between the gut microbiome and HIV reservoir remains inconclusive.

The gut microbiome may influence the host’s immune response *via* their immunoregulatory metabolites or peptides. Moreover, gut microbes can also pass the intestinal barrier and translocate into the systematic circulation, thereby triggering an immune response (Brenchley et al., 2004; Mattapallil et al., 2005; Mudd and Brenchley, 2016). Indeed, microbial translocation has previously been observed in PLHIV (Nganou-Makamdop et al., 2021). It was observed that translocated *Serratia* genera in blood drive innate and Th17 cytokine responses and associate with reduced gut barrier integrity (Nganou-Makamdop et al., 2021). We compared the genera and species enriched in the PLHIV in our study with previously reported translocated microbes in PLHIV, such as *Serratia*, *Acidovorax*, *Sphingobium*, and *Burkholderia* genera. However, abundances of these blood genera were either not detected in the fecal samples or not associated with HIV-related phenotypes and immune response in our HIV cohort. Specifically, we did not detect *Serratia* genera in our cohort, potentially because *Serratia* genera tend to colonize the respiratory and urinary tracts rather than the GI tract (Mahlen, 2011).

We further detected two distinct *P. copri* strains between PLHIV and HCs. Interestingly, the control-related strain showed stronger negative associations with cytokine production capacity than the PLHIV-related strain in PLHIV, particularly for IL-6 and IL-10 production (**Figures 5A, B**). A large proportion of PLHIV seem to have lost this control-related *P. copri* strain and show higher levels of IL-6 and IL-10 production. IL-6 and IL-10 both play a critical role in HIV pathogenesis and are used as markers of disease progression (Freeman et al., 2016). For example, a higher level of plasma IL-6 has been linked with increased morbidity and mortality and the failure to reconstitute CD4 counts (Rose-John et al., 2017). Higher PBMC production of IL-10 is linked to low CD4 counts (Vujkovic-Cvijin et al., 2013), and plasma IL-10 levels positively correlate with viral load (Chehimi et al., 1996). The association between *P. copri* strain and cytokine production may be due to specific *P. copri* immunogenic epitopes. Some *P. copri* immunogenic epitope peptides have been shown to induce IFN-γ production of T cells (Pianta et al., 2021), and the control-related, immune-relevant *P. copri* strain identified in the current study indeed showed an enrichment of immunogenic epitopes (**Figure 5E**). Furthermore, the altered *P. copri* strain may also show different microbial translation and different local interactions with the intestinal immune system. *P. copri* can even translocate to the systematic circulation and cause infection (Posteraro et al., 2019). Immunoglobulin G (IgG) specific to *Prevotella* species including *Prevotella intermedia* and *Prevotella gingivalis* has been found in circulation in patients with rheumatoid arthritis (Mikuls et al., 2012). However, more studies are required to understand the functionality differences between these two strains we identified.

We acknowledge several limitations of the current study. Although it is, to date, a large metagenomics-based study in PLHIV, the sample size is still relatively small. The analysis power can be small after correcting the number of tests performed. Another limitation of this study is the potential batch effects among the different cohorts due to non-biological factors such as technical differences. We also did not have an independent cohort of PLHIV to validate our findings, but we could replicate the DI and FI scores in the 500FG cohort (built in the same medical center and used the same sample collection method as the PLHIV cohort). In addition, we focus on the PLHIV-associated bacterial changes observed in PLHIV who are mostly MSM. Hence, our conclusions may not be generalizable to all PLHIV. However, because of the majority of PLHIV in Europe is MSM, it is still valuable to explore this PLHIV-associated bacterial changes that combined the effects of HIV infection and sexual behavior together. Another potential confounding effect would be related to disease comorbidity. PLHIV show a higher risk for various diseases, particularly cardiometabolic diseases. In our PLHIV cohort, 18.9% of patients have hypertension, 24.5% have mental problems, and 7% have diabetes. However, HCs were only matched on age, sex, and BMI, so we cannot exclude that the observed gut dysbiosis was due to disease comorbidities. We also acknowledge that IL-10 production data were not available for the HCs, so we could not compare changes in IL-10 production capacity between PLHIV and HCs. Furthermore, no blood microbiome data are available for our cohort; thus, we cannot directly assess microbial translocation in PLHIV and to what extent gut microbes can be translocated into the systematic circulation and thereby affect the host’s immune response. Finally, ethnicity is also an important factor associated with gut microbial composition in PLHIV (Noguera-Julian et al., 2016). All PLHIV in our cohort have European ancestry. Hence, in the future study, we suggested collecting samples from across different ethnicities and controlling for its confounding effect. For the technical side, DNA isolation methods can also bias microbial profiling, making microbial data not comparable across different studies. The current study employed the FSK protocol that included a heating step in combination with the enzymatic lysis. Although previous studies have described that this cell lysis method can favor bacterial cell lysis by denaturizing the membrane proteins, this protocol seemed to yield a lower DNA concentration and a microbial community with a lower diversity, compared with DNA isolation method using a bead-beating step as the mechanical lysis (Fernández-Pato et al., 2022).

In conclusion, we observed differential microbial composition and function on the species and strain levels in long-term treated PLHIV. This HIV-associated bacterial signature was linked with HIV reservoir parameters and with PBMC production capacity of IL-1β and IL-10. A large fraction of the PLHIV have lost the control-related *P. copri* strain that was associated with IL-6 and IL-10 production capacity and with CD4 counts and CD4-recovery-abs. The loss of this control-related strain may contribute to a higher level of IL-6 and IL-10 production in PLHIV and to later immune activation and dysfunction. Our observation has provided deeper insight into the critical role of the gut microbiome during HIV infection. The immune-related *P. copri* strain may be used as part of treatment for chronic inflammation, particularly cytokine imbalance; however, more studies are warranted.

## Data availability statement

Raw metagenomic sequencing data of HIV cohort are publicly available from SRA under project number PRJNA820547. Raw metagenomic sequencing data of Dutch Microbiome Project (DMP) and 500 Functional Genomics (500FG) cohort are publicly available from the European Genome-Phenome Archive via accession number EGAS00001005027 and from NCBI Short Read Archive (SRA) via accession number PRJNA942468, respectively. The code used for statistical analysis is available via GitHub: https://github.com/White-Shinobi/HIV-and-gut-microbiome.

## Ethics statement

The 200HIV study was approved by the Medical Ethical ReviewCommittee region Arnhem-Nijmegen (CMO2012-550). For theDutch Microbiome Project (DMP) cohort, the Lifelines study wasapproved by the medical ethical committee from the UniversityMedical Center Groningen (METc number: 2017/152). The 500Functional Genomics (500FG) study was approved by the EthicalCommittee of Radboud University Nijmegen (NL42561.091.12,2012/550). All informed consents were collected for allparticipants. Experiments were conducted in accordance with theprinciples of the Declaration of Helsinki.

## Author contributions

JF and AV conceptualized and managed the study. WH, LV, QM, LJ, RW, AZ, and MN contributed to data generation. YZ, SA-S, DW, and RG analyzed the data. YZ, JF, and AV drafted the manuscript. SA-S, NV, DW, VM, WH, RG, RW, AZ, LV, QM, LJ, and MN reviewed and edited the manuscript. All authors contributed to the article and approved the submitted version.

## Glossary

**Table:**

PLHIV	people living with HIV
ART	antiretroviral therapy
HIV	human immunodeficiency virus
LPS	lipopolysaccharide
DMP	Dutch Microbiome Project
HC	healthy control
BMI	body mass index
MSM	men who have sex with men
SCFA	short-chain fatty acid
FDR	false discovery rate
RAI	receptive anal intercourse
500FG project	500 Functional Genomics Project
NO	nitric oxide
TCA cycle	tricarboxylic acid cycle
P/B ratio	*Prevotella*-to-*Bacteroides* ratio
DI score	dysbiosis index score
FI score	function imbalance score
CD4^+^ T-cell–associated HIV-1 DNA	CA-HIV-DNA
CD4^+^ T-cell–associated HIV-1 RNA	CA-HIV-RNA
Num-P	the number of sexual partners in the previous year
SO	sexual orientation
CD4-recovery-abs	CD4^+^ T-cell absolute recovery level after cART
IEDB	Immune Epitope Database and Analysis Resource
GI	gastrointestinal.

## References

[B1] Armstrong A. J. S. Shaffer M. Nusbacher N. M. Griesmer C. Fiorillo S. Schneider J. M.. (2018). An exploration of Prevotella-rich microbiomes in HIV and men who have sex with men. Microbiome 6, 198. doi: 10.1186/s40168-018-0580-7 30396369 PMC6219090

[B2] Baumgartner M. R. Hu C. A. Almashanu S. Steel G. Obie C. Aral B.. (2000). Hyperammonemia with reduced ornithine, citrulline, arginine and proline: a new inborn error caused by a mutation in the gene encoding delta(1)-pyrroline-5-carboxylate synthase. Hum. Mol. Genet. 9, 2853–2858. doi: 10.1093/hmg/9.19.2853 11092761

[B3] Beghini F. McIver L. J. Blanco-Míguez A. Dubois L. Asnicar F. Maharjan S.. (2021). Integrating taxonomic, functional, and strain-level profiling of diverse microbial communities with biobakery 3. Elife 10. doi: 10.7554/eLife.65088 PMC8096432 33944776

[B4] Brenchley J. M. Schacker T. W. Ruff L. E. Price D. A. Taylor J. H. Beilman G. J.. (2004). CD4+ T cell depletion during all stages of HIV disease occurs predominantly in the gastrointestinal tract. J. Exp. Med. 200, 749–759. doi: 10.1084/jem.20040874 15365096 PMC2211962

[B5] Chang Y.-J. Pukall R. Saunders E. Lapidus A. Copeland A. Nolan M.. (2010). Complete genome sequence of Acidaminococcus fermentans type strain (VR4). Stand. Genomic Sci. 3, 1–14. doi: 10.4056/sigs.1002553 21304687 PMC3035267

[B6] Chehimi J. Ma X. Chouaib S. Zyad A. Nagashunmugam T. Wojcik L.. (1996). Differential production of interleukin 10 during human immunodeficiency virus infection. AIDS Res. Hum. Retroviruses 12, 1141–1149. doi: 10.1089/aid.1996.12.1141 8844018

[B7] Churchill M. J. Deeks S. G. Margolis D. M. Siliciano R. F. Swanstrom R. (2016). HIV reservoirs: what, where and how to target them. Nat. Rev. Microbiol. 14, 55–60. doi: 10.1038/nrmicro.2015.5 26616417

[B8] Connolly N. C. Riddler S. A. Rinaldo C. R. (2005). Proinflammatory cytokines in HIV disease-a review and rationale for new therapeutic approaches. AIDS Rev. 7, 168–180. 16302465

[B9] Crakes K. R. Jiang G. (2019). Gut microbiome alterations during HIV/SIV infection: implications for HIV cure. Front. Microbiol. 10. doi: 10.3389/fmicb.2019.01104 PMC6539195 31191468

[B10] Daillère R. Vétizou M. Waldschmitt N. Yamazaki T. Isnard C. Poirier-Colame V.. (2016). Enterococcus hirae and Barnesiella intestinihominis Facilitate Cyclophosphamide-Induced Therapeutic Immunomodulatory Effects. Immunity 45, 931–943. doi: 10.1016/j.immuni.2016.09.009 27717798

[B11] Dillon S. M. Kibbie J. Lee E. J. Guo K. Santiago M. L. Austin G. L.. (2017). Low abundance of colonic butyrate-producing bacteria in HIV infection is associated with microbial translocation and immune activation. AIDS 31, 511–521. doi: 10.1097/QAD.0000000000001366 28002063 PMC5263163

[B12] Dillon S. M. Lee E. J. Donovan A. M. Guo K. Harper M. S. Frank D. N.. (2016). Enhancement of HIV-1 infection and intestinal CD4+ T cell depletion ex vivo by gut microbes altered during chronic HIV-1 infection. Retrovirology 13, 5. doi: 10.1186/s12977-016-0237-1 26762145 PMC4712466

[B13] Dillon S. M. Lee E. J. Kotter C. V. Austin G. L. Dong Z. Hecht D. K.. (2014). An altered intestinal mucosal microbiome in HIV-1 infection is associated with mucosal and systemic immune activation and endotoxemia. Mucosal Immunol. 7, 983–994. doi: 10.1038/mi.2013.116 24399150 PMC4062575

[B14] Dillon S. M. Manuzak J. A. Leone A. K. Lee E. J. Rogers L. M. McCarter M. D.. (2012). HIV-1 infection of human intestinal lamina propria CD4+ T cells in *vitro* is enhanced by exposure to commensal Escherichia coli. J. Immunol. 189, 885–896. doi: 10.4049/jimmunol.1200681 22689879 PMC3395168

[B15] Dirajlal-Fargo S. Alam K. Sattar A. Kulkarni M. Funderburg N. Wilson W. H.. (2017). Comprehensive assessment of the arginine pathway and its relationship to inflammation in HIV. AIDS 31, 533–537. doi: 10.1097/QAD.0000000000001363 27922857 PMC5263146

[B16] El-Far M. Durand M. Turcotte I. Larouche-Anctil E. Sylla M. Zaidan S.. (2021). Upregulated IL-32 expression and reduced gut short chain fatty acid caproic acid in people living with HIV with subclinical atherosclerosis. Front. Immunol. 12. doi: 10.3389/fimmu.2021.664371 PMC8083984 33936102

[B17] Fernández-Pato A. Sinha T. Gacesa R. Gois M. F. B. Gelderloos-Arends J. Jansen D. B. H.. (2022). Choice of DNA extraction method affects stool microbiome recovery and subsequent phenotypic association analyses. doi: 10.21203/rs.3.rs-1967940/v1 PMC10873414 38366085

[B18] Finn R. D. Mistry J. Tate J. Coggill P. Heger A. Pollington J. E.. (2010). The Pfam protein families database. Nucleic Acids Res. 38, D211–D222. doi: 10.1093/nar/gkp985 19920124 PMC2808889

[B19] Frankel A. E. Deshmukh S. Reddy A. Lightcap J. Hayes M. McClellan S.. (2019). Cancer immune checkpoint inhibitor therapy and the gut microbiota. Integr. Cancer Ther. 18, 1534735419846379. doi: 10.1177/1534735419846379 31014119 PMC6482659

[B20] Freeman M. L. Shive C. L. Nguyen T. P. Younes S.-A. Panigrahi S. Lederman M. M. (2016). Cytokines and T-cell homeostasis in HIV infection. J. Infect. Dis. 214 Suppl, S51–S57. doi: 10.1093/infdis/jiw287 27625431 PMC6373575

[B21] Gacesa R. Kurilshikov A. Vich Vila A. Sinha T. Klaassen M. A. Y. Bolte L. A.. (2022). Environmental factors shaping the gut microbiome in a Dutch population. Nature 604 (7907), 732–739. doi: 10.1038/s41586-022-04567-7 35418674

[B22] Gootenberg D. B. Paer J. M. Luevano J.-M. Kwon D. S. (2017). HIV-associated changes in the enteric microbial community: potential role in loss of homeostasis and development of systemic inflammation. Curr. Opin. Infect. Dis. 30, 31–43. doi: 10.1097/QCO.0000000000000341 27922852 PMC5325247

[B23] Gosmann C. Anahtar M. N. Handley S. A. Farcasanu M. Abu-Ali G. Bowman B. A.. (2017). Lactobacillus-deficient cervicovaginal bacterial communities are associated with increased HIV acquisition in young South African women. Immunity 46, 29–37. doi: 10.1016/j.immuni.2016.12.013 28087240 PMC5270628

[B24] Hileman C. O. Funderburg N. T. (2017). Inflammation, immune activation, and antiretroviral therapy in HIV. Curr. HIV/AIDS Rep. 14, 93–100. doi: 10.1007/s11904-017-0356-x 28434169 PMC5514315

[B25] Ishizaka A. Koga M. Mizutani T. Parbie P. K. Prawisuda D. Yusa N.. (2021). Unique gut microbiome in HIV patients on antiretroviral therapy (ART) suggests association with chronic inflammation. Microbiol. Spectr. 9, e0070821. doi: 10.1128/Spectrum.00708-21 34378948 PMC8552706

[B26] Kak G. Raza M. Tiwari B. K. (2018). Interferon-gamma (IFN-γ): Exploring its implications in infectious diseases. Biomol. Concepts 9, 64–79. doi: 10.1515/bmc-2018-0007 29856726

[B27] Kaminski J. Gibson M. K. Franzosa E. A. Segata N. Dantas G. Huttenhower C. (2015). High-specificity targeted functional profiling in microbial communities with shortBRED. PloS Comput. Biol. 11, e1004557. doi: 10.1371/journal.pcbi.1004557 26682918 PMC4684307

[B28] Keating S. M. Jacobs E. S. Norris P. J. (2012). Soluble mediators of inflammation in HIV and their implications for therapeutics and vaccine development. Cytokine Growth Factor Rev. 23, 193–206. doi: 10.1016/j.cytogfr.2012.05.006 22743035 PMC3418433

[B29] Klatt N. R. Chomont N. Douek D. C. Deeks S. G. (2013). Immune activation and HIV persistence: implications for curative approaches to HIV infection. Immunol. Rev. 254, 326–342. doi: 10.1111/imr.12065 23772629 PMC3694608

[B30] Ling Z. Jin C. Xie T. Cheng Y. Li L. Wu N. (2016). Alterations in the fecal microbiota of patients with HIV-1 infection: an observational study in A Chinese population. Sci. Rep. 6, 30673. doi: 10.1038/srep30673 27477587 PMC4967929

[B31] Lozupone C. A. Li M. Campbell T. B. Flores S. C. Linderman D. Gebert M. J.. (2013). Alterations in the gut microbiota associated with HIV-1 infection. Cell Host Microbe 14, 329–339. doi: 10.1016/j.chom.2013.08.006 24034618 PMC3864811

[B32] Lu W. Feng Y. Jing F. Han Y. Lyu N. Liu F.. (2018). Association between gut microbiota and CD4 recovery in HIV-1 infected patients. Front. Microbiol. 9. doi: 10.3389/fmicb.2018.01451 PMC6043814 30034377

[B33] Mahlen S. D. (2011). Serratia infections: from military experiments to current practice. Clin. Microbiol. Rev. 24, 755–791. doi: 10.1128/CMR.00017-11 21976608 PMC3194826

[B34] Mattapallil J. J. Douek D. C. Hill B. Nishimura Y. Martin M. Roederer M. (2005). Massive infection and loss of memory CD4+ T cells in multiple tissues during acute SIV infection. Nature 434, 1093–1097. doi: 10.1038/nature03501 15793563

[B35] Mikuls T. R. Thiele G. M. Deane K. D. Payne J. B. O’Dell J. R. Yu F.. (2012). Porphyromonas gingivalis and disease-related autoantibodies in individuals at increased risk of rheumatoid arthritis. Arthritis Rheumatol. 64, 3522–3530. doi: 10.1002/art.34595 PMC3467347 22736291

[B36] Mudd J. C. Brenchley J. M. (2016). Gut mucosal barrier dysfunction, microbial dysbiosis, and their role in HIV-1 disease progression. J. Infect. Dis. 214 Suppl, S58–S66. doi: 10.1093/infdis/jiw258 27625432 PMC5021240

[B37] Murray M. F. (2003). Tryptophan depletion and HIV infection: a metabolic link to pathogenesis. Lancet Infect. Dis. 3, 644–652. doi: 10.1016/s1473-3099(03)00773-4 14522263

[B38] Nganou-Makamdop K. Talla A. Sharma A. A. Darko S. Ransier A. Laboune F.. (2021). Translocated microbiome composition determines immunological outcome in treated HIV infection. Cell 184, 3899–3914.e16. doi: 10.1016/j.cell.2021.05.023 34237254 PMC8316372

[B39] Noguera-Julian M. Rocafort M. Guillén Y. Rivera J. Casadellà M. Nowak P.. (2016). Gut microbiota linked to sexual preference and HIV infection. EBioMedicine 5, 135–146. doi: 10.1016/j.ebiom.2016.01.032 27077120 PMC4816837

[B40] Nowak P. Troseid M. Avershina E. Barqasho B. Neogi U. Holm K.. (2015). Gut microbiota diversity predicts immune status in HIV-1 infection. AIDS 29, 2409–2418. doi: 10.1097/QAD.0000000000000869 26355675

[B41] Osuji F. N. Onyenekwe C. C. Ahaneku J. E. Ukibe N. R. (2018). The effects of highly active antiretroviral therapy on the serum levels of pro-inflammatory and anti-inflammatory cytokines in HIV infected subjects. J. Biomed. Sci. 25, 88. doi: 10.1186/s12929-018-0490-9 30501642 PMC6276218

[B42] Papadia C. Kelly P. Caini S. Roberto Corazza G. Shawa T. Franzè A.. (2010). Plasma citrulline as a quantitative biomarker of HIV-associated villous atrophy in a tropical enteropathy population. Clin. Nutr. 29, 795–800. doi: 10.1016/j.clnu.2010.04.008 20646802

[B43] Parada Venegas D. de la Fuente M. K. Landskron G. González M. J. Quera R. Dijkstra G.. (2019). Short chain fatty acids (SCFAs)-mediated gut epithelial and immune regulation and its relevance for inflammatory bowel diseases. Front. Immunol. 10. doi: 10.3389/fimmu.2019.00277 PMC6421268 30915065

[B44] Pianta A. Chiumento G. Ramsden K. Wang Q. Strle K. Arvikar S.. (2021). Identification of novel, immunogenic HLA-DR-presented prevotella copri peptides in patients with rheumatoid arthritis. Arthritis Rheumatol. (Hoboken N.J.) 73, 2200–2205. doi: 10.1002/art.41807 PMC8626540 34042327

[B45] Pinto-Cardoso S. Lozupone C. Briceño O. Alva-Hernández S. Téllez N. Adriana A.. (2017). Fecal Bacterial Communities in treated HIV infected individuals on two antiretroviral regimens. Sci. Rep. 7, 43741. doi: 10.1038/srep43741 28262770 PMC5338340

[B46] Planès R. Serrero M. Leghmari K. BenMohamed L. Bahraoui E. (2018). HIV-1 envelope glycoproteins induce the production of TNF-α and IL-10 in human monocytes by activating calcium pathway. Sci. Rep. 8, 17215. doi: 10.1038/s41598-018-35478-1 30464243 PMC6249280

[B47] Posteraro P. De Maio F. Menchinelli G. Palucci I. Errico F. M. Carbone M.. (2019). First bloodstream infection caused by Prevotella copri in a heart failure elderly patient with Prevotella-dominated gut microbiota: a case report. Gut Pathog. 11, 44. doi: 10.1186/s13099-019-0325-6 31548871 PMC6749625

[B48] Ray S. Narayanan A. Giske C. G. Neogi U. Sönnerborg A. Nowak P. (2021). Altered gut microbiome under antiretroviral therapy: impact of efavirenz and zidovudine. ACS Infect. Dis. 7, 1104–1115. doi: 10.1021/acsinfecdis.0c00536 33346662 PMC8154435

[B49] Rose-John S. Winthrop K. Calabrese L. (2017). The role of IL-6 in host defence against infections: immunobiology and clinical implications. Nat. Rev. Rheumatol. 13, 399–409. doi: 10.1038/nrrheum.2017.83 28615731

[B50] Rutsaert S. De Spiegelaere W. De Clercq L. Vandekerckhove L. (2019). Evaluation of HIV-1 reservoir levels as possible markers for virological failure during boosted darunavir monotherapy. J. Antimicrob. Chemother. 74, 3030–3034. doi: 10.1093/jac/dkz269 31314108

[B51] Schirmer M. Smeekens S. P. Vlamakis H. Jaeger M. Oosting M. Franzosa E. A.. (2016). Linking the human gut microbiome to inflammatory cytokine production capacity. Cell 167, 1897. doi: 10.1016/j.cell.2016.11.046 27984736

[B52] Scholz M. Ward D. V. Pasolli E. Tolio T. Zolfo M. Asnicar F.. (2016). Strain-level microbial epidemiology and population genomics from shotgun metagenomics. Nat. Methods 13, 435–438. doi: 10.1038/nmeth.3802 26999001

[B53] Schröder C. Matthies A. Engst W. Blaut M. Braune A. (2013). Identification and expression of genes involved in the conversion of daidzein and genistein by the equol-forming bacterium Slackia isoflavoniconvertens. Appl. Environ. Microbiol. 79, 3494–3502. doi: 10.1128/AEM.03693-12 23542626 PMC3648055

[B54] Smith E. Morowitz H. J. (2004). Universality in intermediary metabolism. Proc. Natl. Acad. Sci. U. S. A. 101, 13168–13173. doi: 10.1073/pnas.0404922101 15340153 PMC516543

[B55] Tuddenham S. Koay W. L. Sears C. (2020). HIV, sexual orientation, and gut microbiome interactions. Dig. Dis. Sci. 65, 800–817. doi: 10.1007/s10620-020-06110-y 32030625 PMC7301749

[B57] van der Heijden W. A. Van de Wijer L. Keramati F. Trypsteen W. Rutsaert S. Horst R.. (2021). Chronic HIV infection induces transcriptional and functional reprogramming of innate immune cells. JCI Insight 6. doi: 10.1172/jci.insight.145928 PMC8119206 33630761

[B56] Van de Wijer L. van der Heijden W. A. Ter Horst R. Jaeger M. Trypsteen W. Rutsaert S.. (2021). The architecture of circulating immune cells is dysregulated in people living with HIV on long term antiretroviral treatment and relates with markers of the HIV-1 reservoir, cytomegalovirus, and microbial translocation. Front. Immunol. 12. doi: 10.3389/fimmu.2021.661990 PMC8091964 33953724

[B58] Vázquez-Castellanos J. F. Serrano-Villar S. Jiménez-Hernández N. Soto Del Rio M. D. Gayo S. Rojo D.. (2018). Interplay between gut microbiota metabolism and inflammation in HIV infection. ISME J. 12, 1964–1976. doi: 10.1038/s41396-018-0151-8 29789624 PMC6052150

[B59] Vázquez-Castellanos J. F. Serrano-Villar S. Latorre A. Artacho A. Ferrús M. L. Madrid N.. (2015). Altered metabolism of gut microbiota contributes to chronic immune activation in HIV-infected individuals. Mucosal Immunol. 8, 760–772. doi: 10.1038/mi.2014.107 25407519

[B60] Vita R. Mahajan S. Overton J. A. Dhanda S. K. Martini S. Cantrell J. R.. (2019). The immune epitope database (IEDB): 2018 update. Nucleic Acids Res. 47, D339–D343. doi: 10.1093/nar/gky1006 30357391 PMC6324067

[B61] Vujkovic-Cvijin I. Dunham R. M. Iwai S. Maher M. C. Albright R. G. Broadhurst M. J.. (2013). Dysbiosis of the gut microbiota is associated with HIV disease progression and tryptophan catabolism. Sci. Transl. Med. 5, 193ra91. doi: 10.1126/scitranslmed.3006438 PMC4094294 23843452

[B62] Vujkovic-Cvijin I. Somsouk M. (2019). HIV and the gut microbiota: composition, consequences, and avenues for amelioration. Curr. HIV/AIDS Rep. 16, 204–213. doi: 10.1007/s11904-019-00441-w 31037552 PMC6579656

[B63] Vujkovic-Cvijin I. Sortino O. Verheij E. Sklar J. Wit F. W. Kootstra N. A.. (2020). HIV-associated gut dysbiosis is independent of sexual practice and correlates with noncommunicable diseases. Nat. Commun. 11, 2448. doi: 10.1038/s41467-020-16222-8 32415070 PMC7228978

[B64] Zagato E. Pozzi C. Bertocchi A. Schioppa T. Saccheri F. Guglietta S.. (2020). Endogenous murine microbiota member Faecalibaculum rodentium and its human homologue protect from intestinal tumour growth. Nat. Microbiol. 5, 511–524. doi: 10.1038/s41564-019-0649-5 31988379 PMC7048616

[B65] Zeybel M. Arif M. Li X. Altay O. Shi M. Akyildiz M.. (2022). Multiomics analysis reveals the impact of microbiota on host metabolism in hepatic steatosis. Adv. Sci. (Weinheim, Baden-Wurttemberg, Germany) 9 (11), e2104373. doi: 10.1002/advs.202104373 PMC9008426 35128832

[B66] Zilberman-Schapira G. Zmora N. Itav S. Bashiardes S. Elinav H. Elinav E. (2016). The gut microbiome in human immunodeficiency virus infection. BMC Med. 14, 83. doi: 10.1186/s12916-016-0625-3 27256449 PMC4891875

