# Supplementary material for: Gut dysbiosis associates with cytokine production capacity in viral-suppressed people living with HIV
Source: Front Cell Infect Microbiol. 2023 Jul 31;13:1202035. doi: 10.3389/fcimb.2023.1202035 (PMC10425223; doi:10.3389/fcimb.2023.1202035)

Supplementary Figures for

Gut dysbiosis associates with cytokine production capacity in viral-suppressed people living with HIV

Zhang et al.

Contents of Supplementary Figures

Figure S1. Comparison of the microbial pathway alpha diversity between people living with HIV (PLHIV) and healthy controls (HCs) from DMP cohort.

Figure S2. Relative abundance of Prevotella species in PLHIV and HCs in DMP cohort.

Figure S3. Barplot plot comparing bacterial changes between 96 PLHIV-MSM vs. 53 DMP-MSM and 143 PLHIV vs. 190 DMP-MSW.

Figure S4. Barplot plot comparing bacterial changes between 96 PLHIV-MSM vs. 53 DMP-MSM and 143 PLHIV vs. 190 DMP-MSW.

Figure S5. Violin plots comparing microbiome indices between the 500FG, DMP and HIV cohorts.

Figure S6. Distribution of HIV-related phenotypes in PLHIV.

Figure S7. Relationships between HIV-related phenotypes.

Figure S8. Heatmap of associations between HIV-associated bacterial signatures (Shannon index, beta diversity, P/B ratio, DI and FI score) and HIV-related phenotypes.

Figure S9. Relationship between HIV-related phenotypes and cytokine production capacity.

Figure S10. Distribution of P. copri strains among people with different sexual orientation in HIV and DMP cohort.

Figure S11. Distinct associations of the relative abundances of the control-related strain and HIV-related Prevotella copri strain with IL-6 (a, b) and TNF production capacity (c) in HCs from 500FG cohort, established using linear regression.

Figure S1. Comparison of the microbial pathway alpha diversity between people living with HIV (PLHIV) and healthy controls (HCs) from DMP cohort. Related to Figure 1. Y-axis is the Shannon index at the bacterial pathway level.

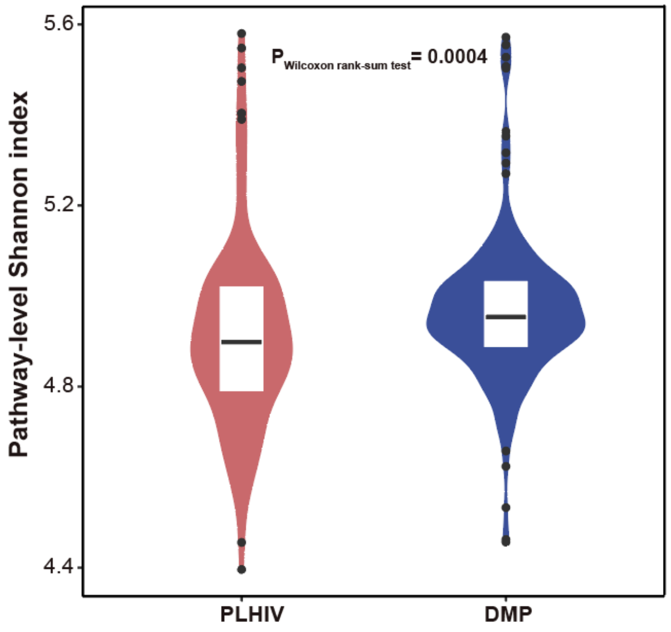

Figure S2. Relative abundance of *Prevotella* species in PLHIV and HCs in DMP cohort. Related to Figure 1. **a.** *Prevotella copri*. **b.** *Prevotella sp 885.* **c.** *Prevotella sp AM42 24*. **d.** *Prevotella sp CAG 1092*. **e.** *Prevotella sp CAG 279*. **f.** *Prevotella sp CAG 520*. **g.** *Prevotella sp CAG 5226*. **h.** *Prevotella stercorea.*

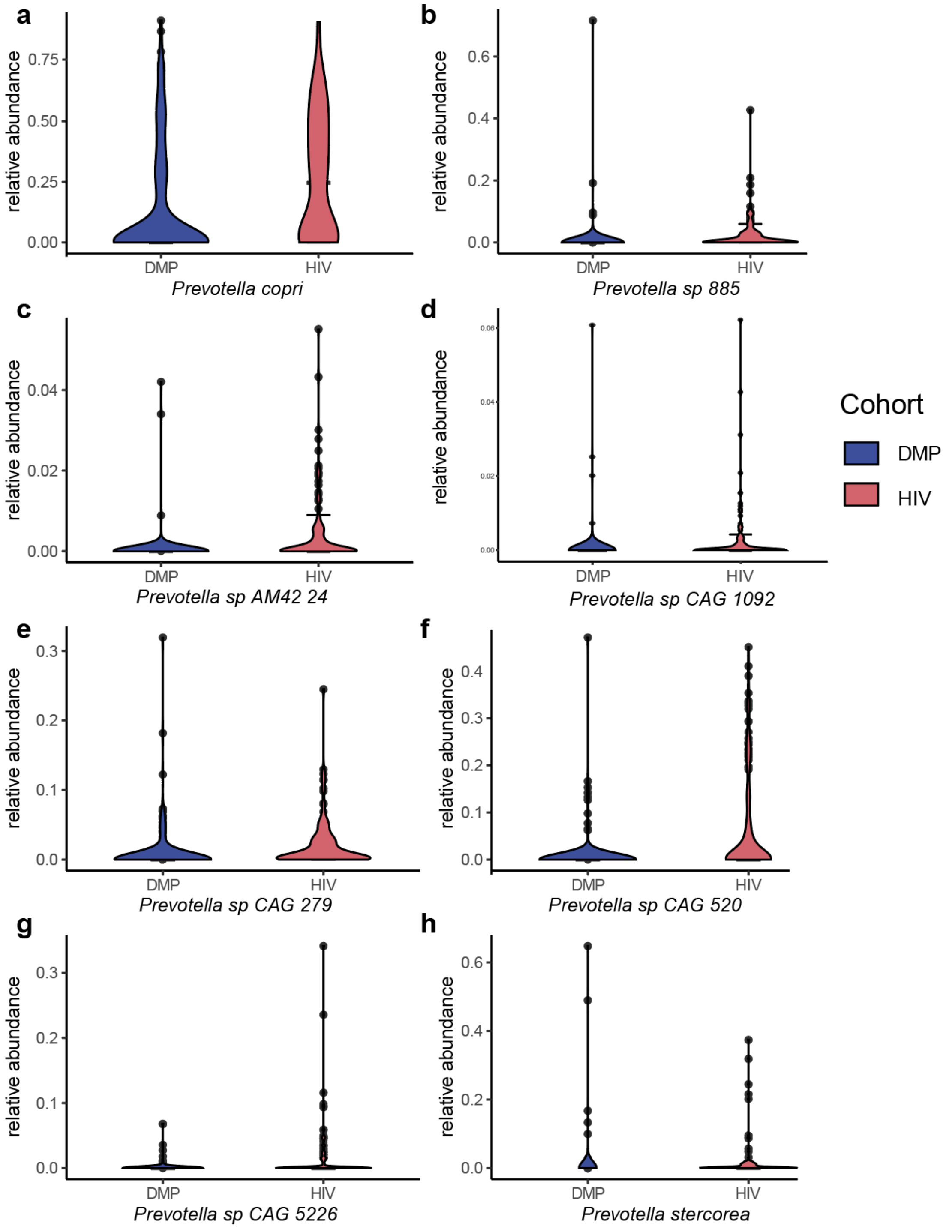

Figure S3. Barplot plot comparing bacterial changes between 96 PLHIV-MSM vs. 53 DMP-MSM and 143 PLHIV vs. 190 DMP-MSW. Out of 76 differentially abundant species between 143 PLHIV vs. 190 DMP-MSW, 51 species (89.5%) were replicated at FDR<0.05 level. MSM: men who have sex with men; MSW: men who have sex with women.

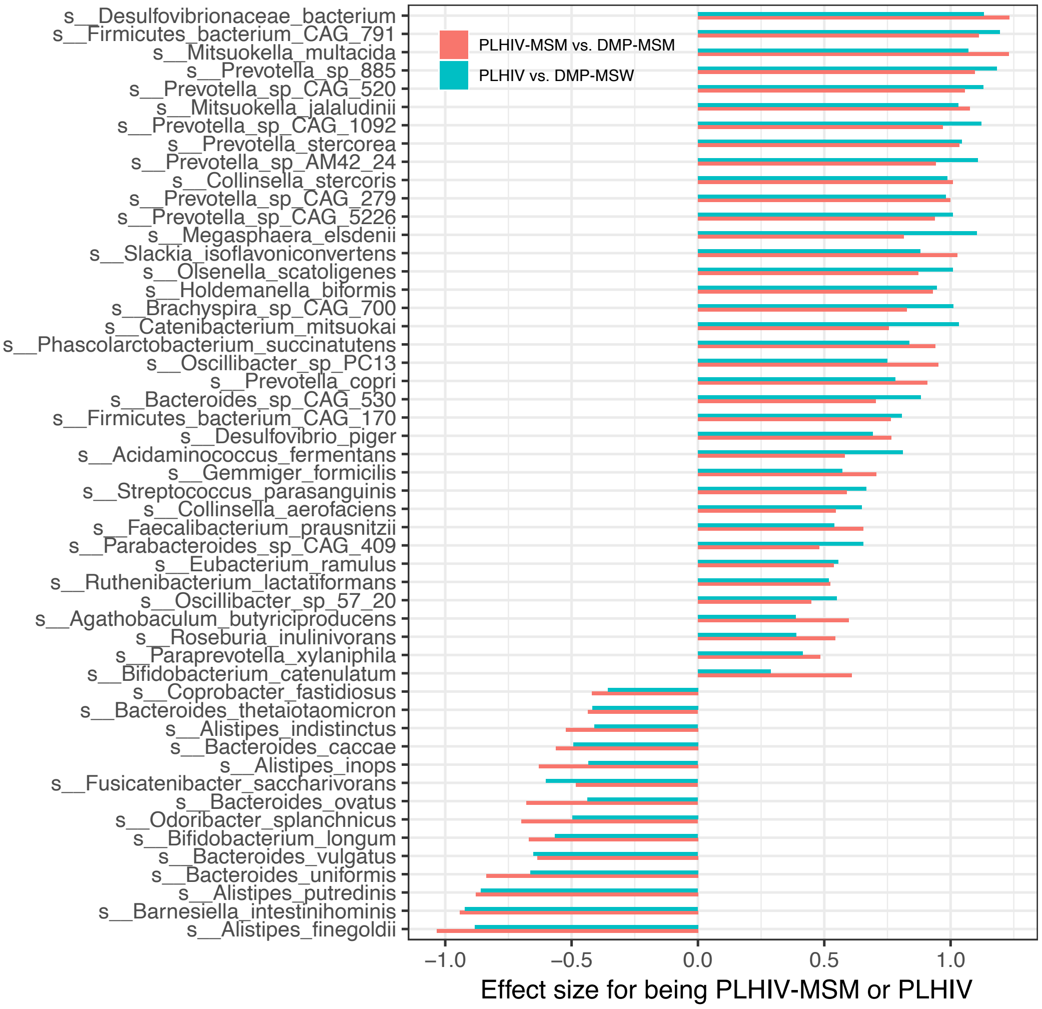

Figure S4. Barplot plot comparing bacterial changes between 96 PLHIV-MSM vs. 53 DMP-MSM and 143 PLHIV vs. 190 DMP-MSW. Out of 163 differentially abundant species between 143 PLHIV vs. 190 DMP-MSW, 102 pathways (62.6%) were replicated at FDR<0.05 level. MSM: men who have sex with men; MSW: men who have sex with women.

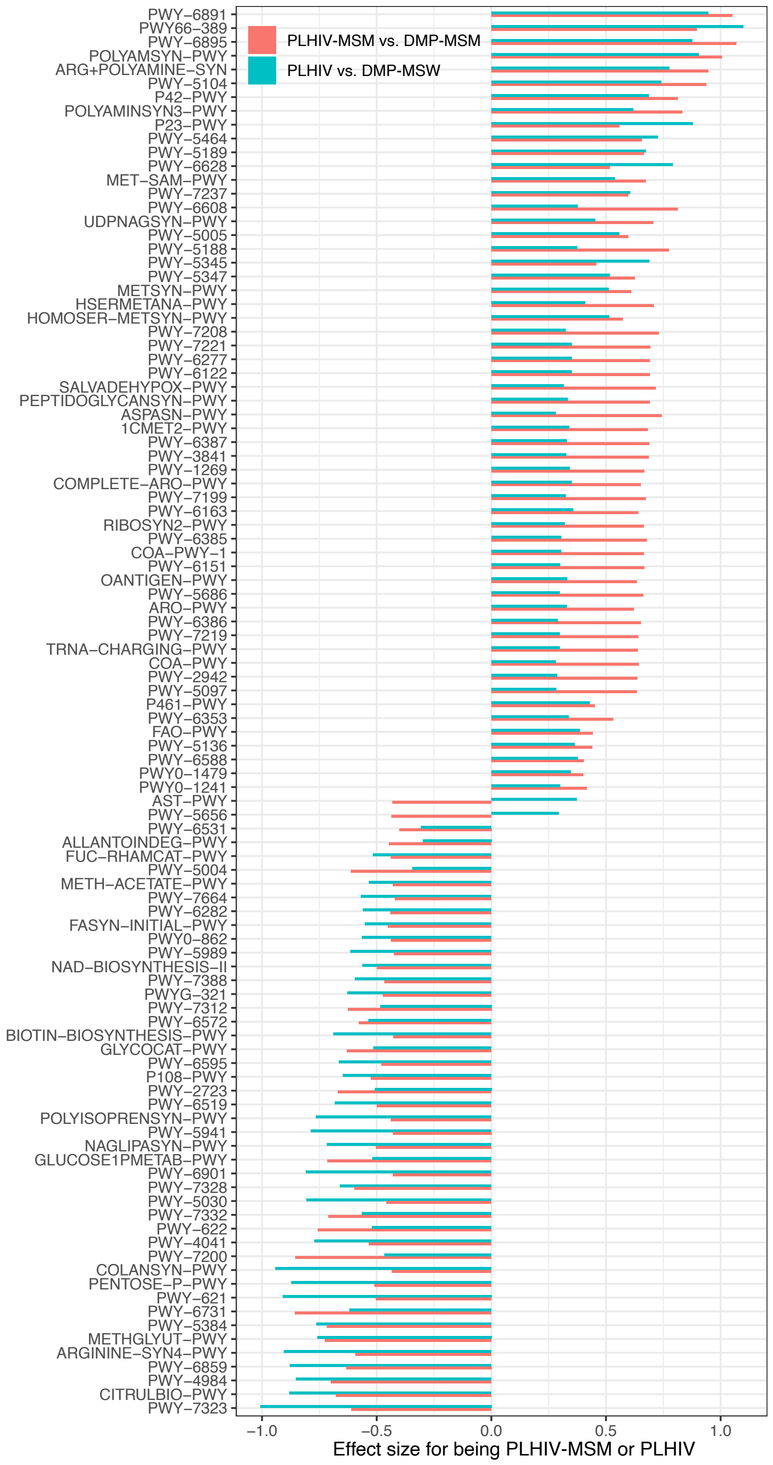

Figure S5. Violin plots comparing microbiome indices between the 500FG, DMP and HIV cohorts. a. Ratio between *Prevotella* and *Bacteroides* genus (P/B). **b.** Dysbiosis index (DI) score. **c.** Functional index (FI) score.

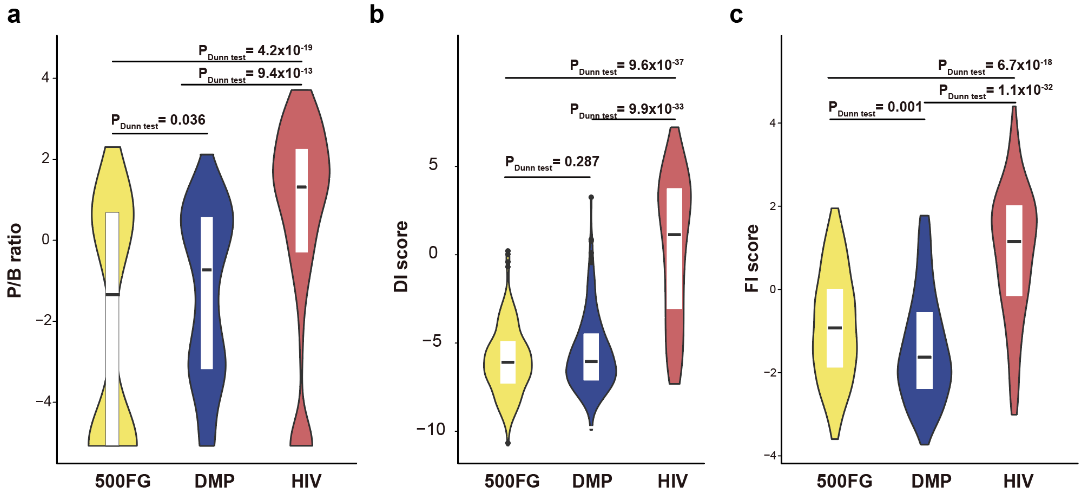

Figure S6. Distribution of HIV-related phenotypes in PLHIV.

a-c. HIV-1 reservoir measurements in circulating CD4$^+$ T cells. **(a)** CD4$^+$ T cell–associated HIV-1 RNA (CA-HIV-RNA), **(b)** CD4$^+$ T cell–associated HIV-1 DNA (CA-HIV-DNA), **(c)** ratio between CA-RNA and CA-DNA (CA-RNA/CA-DNA). **d-h.** CD4$^+$ T cell counts. **(d)** CD4$^+$ T cell counts nadir (CD4 nadir), **(e)** CD4$^+$ T cell counts latest (CD4 counts), **(f)** ratio between CD4$^+$ T cell count and CD8$^+$ T cell counts (CD4/CD8), **(g)** CD4$^+$ cell recovery absolute value (CD4-recovery-abs), **(h)** CD4$^+$ cell recovery relative value (CD4-recovery-re). **i.** Sexual orientation data and receptive anal intercourse (RAI) data. **j-k.** Plasma viral load. **(j)** Zenith value of HIV RNA load level in plasma (HIV RNA zenith), **(k)** HIV RNA level in plasma (HIV RNA load). **l-n.** Duration related to infection and treatment. **(l)** Duration of HIV infection (HIV duration), **(m)** Duration of combined antiretroviral therapy (cART duration), **(n)** Duration between diagnosis of HIV and the start of cART (Time diagnosis-cART). Y-axis in A–H and J–N refers to the count of the phenotypes.

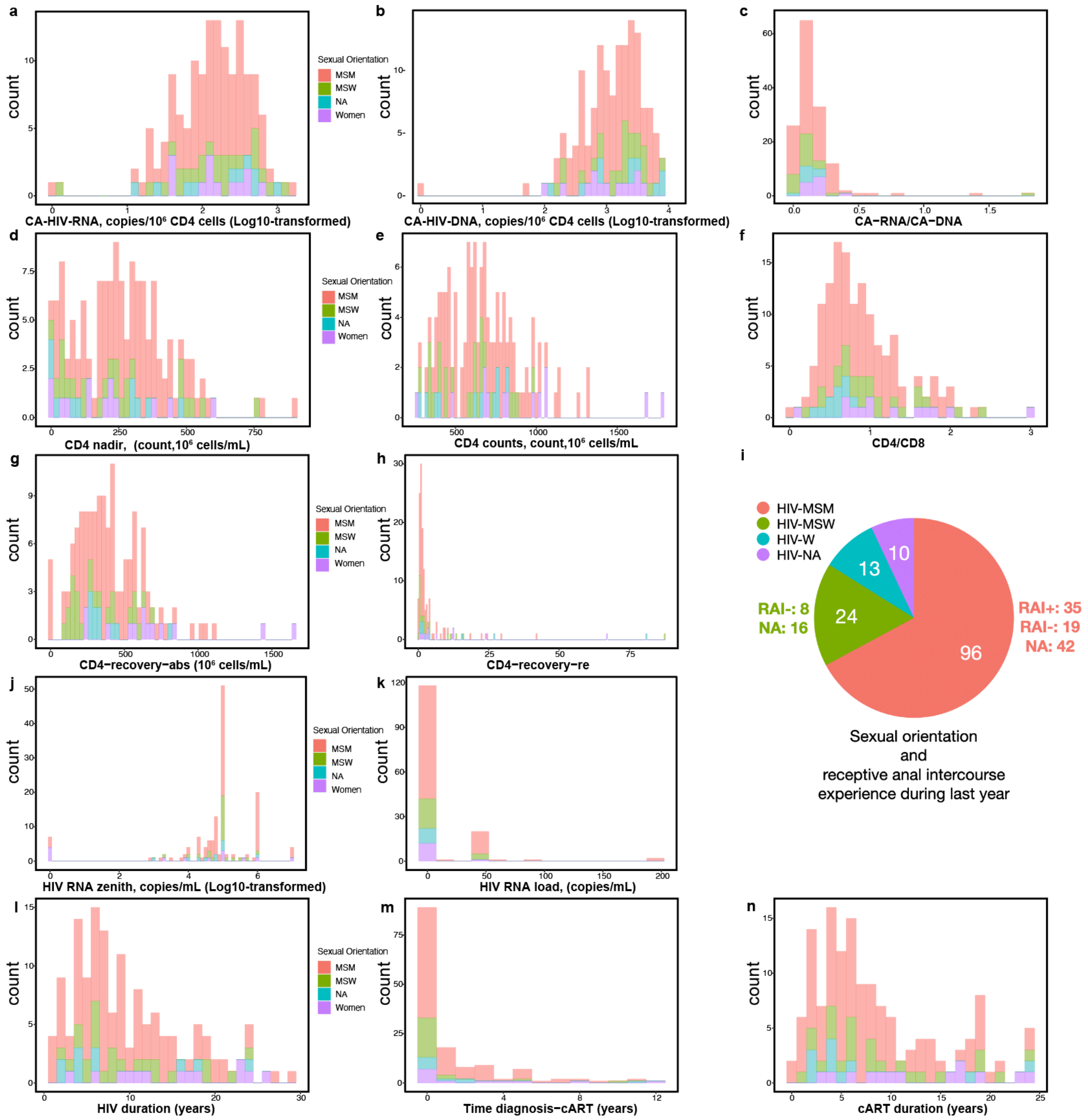

Figure S7. Relationships between HIV-related phenotypes. Heatmap shows the Spearman correlation rho between HIV-related phenotypes. Box color indicates the correlation rho. White boxes indicate an FDR value for the correlation > 0.1.

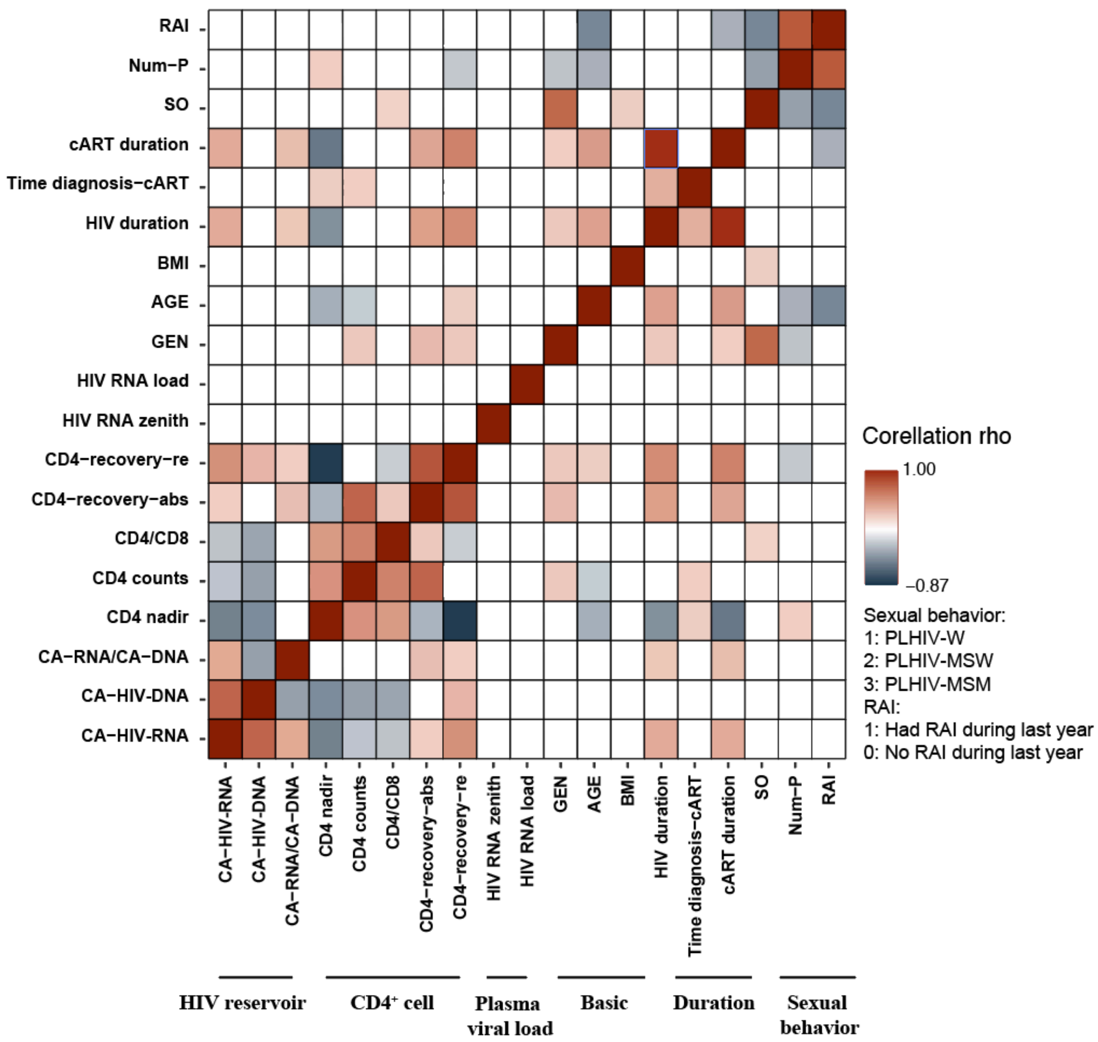

Figure S8. Heatmap of associations between HIV-associated bacterial signatures (Shannon index, beta diversity, P/B ratio, DI and FI score) and HIV-related phenotypes. Heatmap shows Spearman correlation rho. HIV-related phenotypes are corrected for age, gender, read count and sexual behavior. Box color indicates the Spearman correlation rho. White box indicates P > 0.05. Bray-Curtis distance of species and then multiplied by ten to rescale.

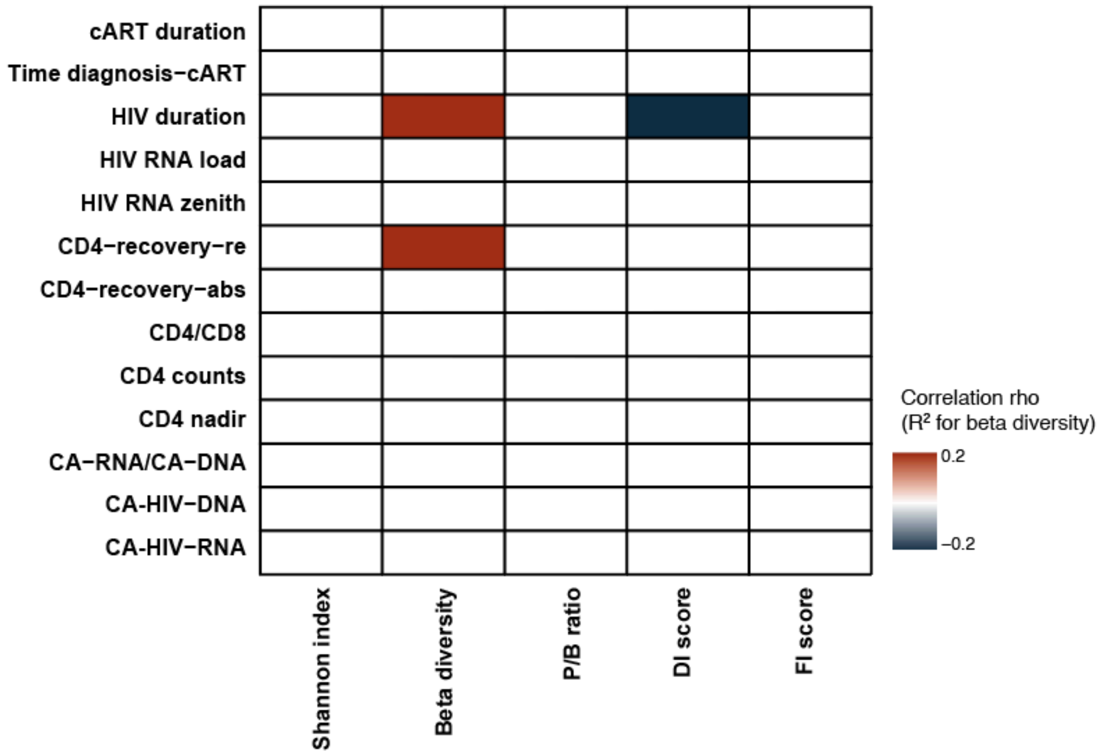

Figure S9. Relationship between HIV-related phenotypes and cytokine production capacity. Heatmap shows the Spearman correlation rho between HIV-related phenotypes and cytokine production capacity. Box colors indicate the correlation rho. White indicates an FDR value for the correlation was > 0.1.

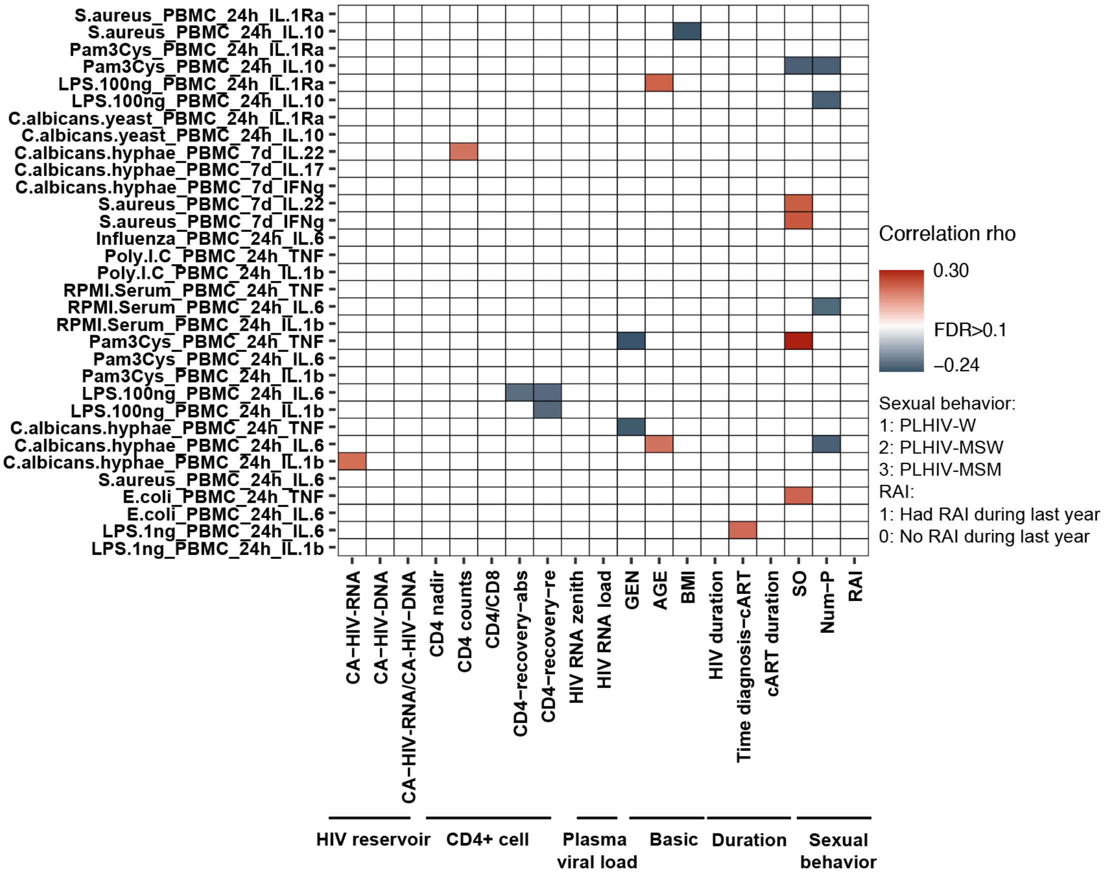

Figure S10. Distribution of *P. copri* strains among people with different sexual orientation in HIV and DMP cohort. MSM: men who have sex with men; MSW, men who have sex with women; WSM, women who have sex with men. Compared with DMP-MSM, PLHIV-MSM showed enrichment in PLHIV-related strain (Fisher exact test, P = 1.1×10^{-4}). Compared with DMP-MSW, DMP-MSM showed enrichment in PLHIV-related strain (Fisher exact test, P = 1.9×10^{-4}). Compared with PLHIV-MSW, PLHIV-MSM also showed enrichment in PLHIV-related strain (Fisher exact test, P = 0.01).

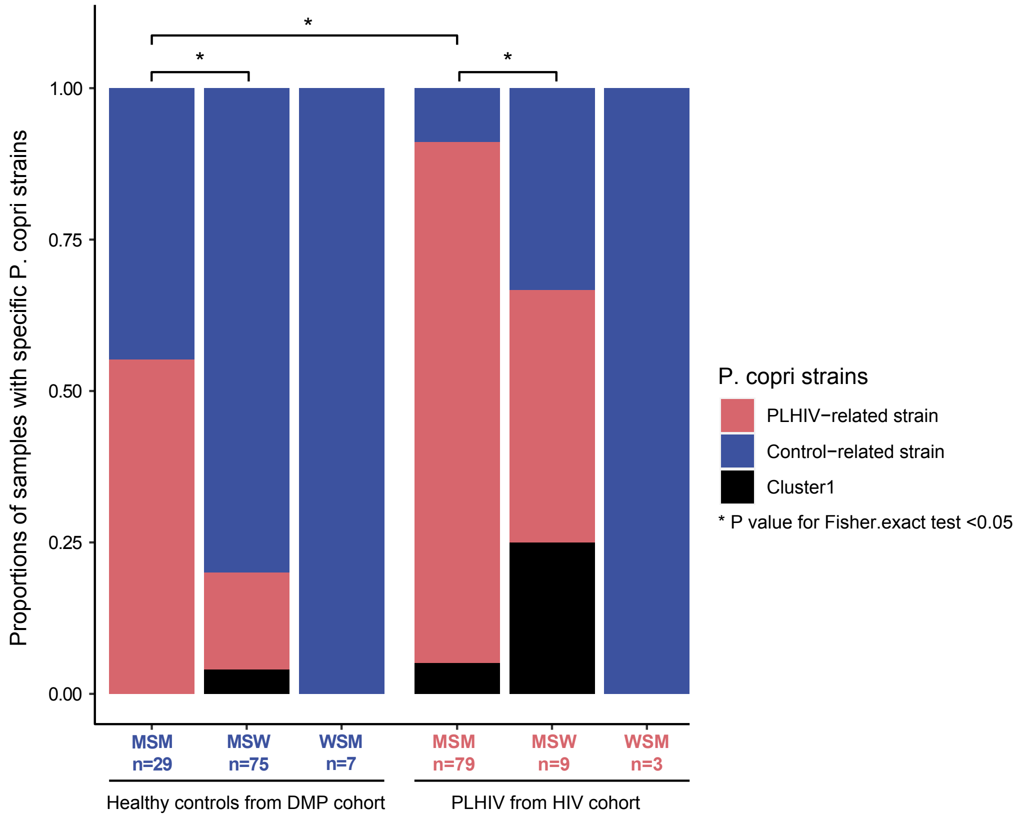

Figure S11. Associations between *P. copri* strains and cytokine production capacity in HCs and PLHIV, established using linear regression. Cytokine production was corrected for age, sex, read counts and sexual behavior. (a) The control-related strain showed the oppositive associations with Pam3Cys-induced IL-6 production between PLHIV and HCs. (b) The PLHIV-related strain showed a negative association with *Candida albicans*-induced IL-10 production, which didn't show heterogeneity against the control-related strain.

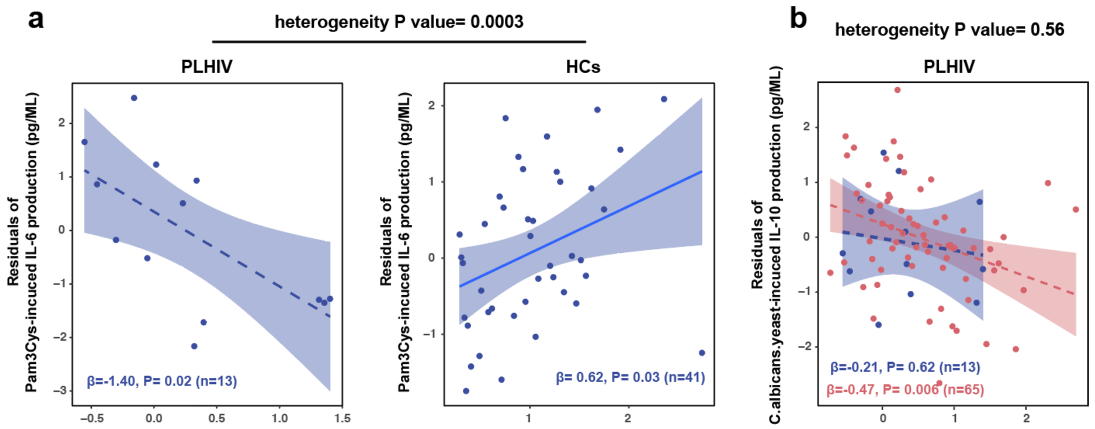

Supplement: Supplementary file 1 [file DataSheet_1.pdf]
